# The effect of STAT1 expression and the Y701 phosphorylation on primary Undifferentiated Pleomorphic Sarcoma cells

**DOI:** 10.1371/journal.pone.0361295

**Published:** 2026-10-26

**Authors:** Victoria S. Coward, Nalan Gokgoz, Kim M. Tsoi, Jay S. Wunder, Irene L. Andrulis

**Affiliations:** 1 Lunenfeld-Tanenbaum Research Institute, Sinai Health System, Toronto, Canada; 2 Department of Molecular Genetics, University of Toronto, Toronto, Canada; 3 Department of Surgical Oncology, Princess Margaret Cancer Centre/Mount Sinai Hospital, Toronto, Canada; 4 Department of Surgery, Mount Sinai Hospital, Sinai Health, Toronto, Canada; 5 University of Toronto Musculoskeletal Oncology Unit, Division of Orthopaedic Surgery, Toronto, Canada; 6 Department of Laboratory Medicine and Pathobiology, University of Toronto, Toronto, Canada; 7 Institute of Medical Science, University of Toronto, Toronto, Canada; University of Zambia School of Medicine, ZAMBIA

## Abstract

Undifferentiated Pleomorphic Sarcoma (UPS) is a genetically complex type of soft tissue sarcoma (STS) and there are few therapeutic options for advanced disease. Immune checkpoint blockade therapy may be of benefit to a subset of UPS patients, particularly those with elevated tumoral Programmed Cell Death Ligand 1 (PD-L1) expression and an inflamed tumour microenvironment. From transcriptomic analyses we previously identified *Signal Transducer and Activator of Transcription 1 (STAT1)* expression, a key mediator of interferon-γ (IFN-γ) signaling, as enriched in a cohort of UPS cases with elevated PD-L1 levels that were observed to have an improved clinical outcome. This led us to investigate the role of STAT1 in UPS biology. We examined the effects of STAT1 loss, and overexpression of either wild type or tyrosine phosphorylation-deficient STAT1 in primary human UPS cell lines. We observed that IFN-γ treatment decreased cell counts, increased cell death, and induced the secretion of CXCR3 ligands in a STAT1-dependent manner. Overexpression of wild type STAT1, but not a tyrosine to phenylalanine phospho-mutant (Y701F), restored IFN-γ-mediated cell death and cytokine secretion, highlighting the importance of Y701 phosphorylation in STAT1-associated antitumour functions. STAT1 Y701 phosphorylation status was associated with gene expression changes that were cell line specific. Together, these findings shed light on the central role of STAT1, and specifically the Y701 phosphorylation, in mediating IFN-γ signaling and related phenotypes. This study underscores the need for further exploration of the role of STAT1 phosphorylation in tumor-immune interactions within UPS.

## Introduction

Soft tissue sarcomas (STS) are a heterogenous group of connective tissue cancers that account for approximately 1% of annual adult cancer diagnoses. Undifferentiated Pleomorphic Sarcomas (UPS) are a genetically complex type of STS that do not have characteristic molecular alterations. Resection with or without radiotherapy is the primary treatment strategy for UPS patients with localized disease however, between 30–50% of patients will metastasize, and then have a poor survival rate [1,2]. Considering this, new treatment strategies are needed.

Within the last decade, a trial of a Programmed Cell Death 1 (PD-1) inhibitor, pembrolizumab, (SARC028) demonstrated that UPS patients were among those that showed a promising response [3]. Additional correlative analyses indicated that SARC028 responders had higher tumoral PD-L1 expression in addition to higher densities of activated T cells at baseline in comparison to non-responders [4]. Our group and others have also found tumoral PD-L1 expression to be related to clinical outcome in UPS [5,6]. We observed that a subset of UPS cases expressed relatively high levels of PD-L1 and increased tumour-infiltrating lymphocytes (TILs) in comparison to cases with low PD-L1 levels. To investigate why some UPS cases expressed PD-L1, were immune infiltrated and had better outcomes while others did not, we used transcriptomic analyses to identify genes uniquely expressed in the UPS PD-L1 high cohort. Th1 pathway genes including those regulated by interferon-gamma (IFN-γ) such as *Signal Transducer and Activator of Transcription 1* (*STAT1*) were identified as enriched in the UPS PD-L1 high cohort [5]. Many Th1-related genes function primarily in immune cells, however STAT1 has been reported to be expressed in both tumour and immune cells.

STAT1 is a transcription factor and member of the well-conserved STAT family that functions in the IFN-γ signaling cascade (reviewed in [7,8]). Upon IFN-γ signalling, STAT1 is canonically phosphorylated on its C-terminal tyrosine (Y701 residue) which has been determined to be required for the full range of IFN-γ signaling [9,10]. IFN-γ-mediated phosphorylated STAT1 (pSTAT1) primarily leads to the formation of STAT1 homodimers that can be translocated to the nucleus. The homodimers recruit histone acetyltransferases and other co-activators to facilitate DNA binding and gene expression. It is important to note that STAT1 can transduce signals from a variety of stimuli and participate in various protein complexes that contribute to distinct transcriptional outputs. For example, phosphorylated STAT1 can also participate in the Interferon-stimulated gene factor 3 (ISGF3) protein complex which includes tyrosine phosphorylated Signal Transducer and Activator of Transcription 2 (STAT2) with Interferon Regulatory Factor 9 (IRF9) and primarily forms downstream of IFN-α/β signaling. Unphosphorylated STATs were previously thought to be inactive however some genes are regulated by STAT complexes that do not require tyrosine phosphorylation. Basal expression of certain genes controlled by unphosphorylated STAT (uSTAT) dimers, such as uSTAT1 homodimers, uSTAT1-uSTAT2 or uSTAT2-IFR9 dimers, has been demonstrated [11–17].

Our understanding of the role of uSTAT1 homodimers and heterodimers in cancer systems is limited. This is in part due to studies focusing on the antiviral activities of IFN-α/β signaling, and due to the pervasive binding nature of STAT2 and uSTAT2 [13]. However, it is important to better understand uSTAT1 and its context-specific functions because it has been associated with negative clinical outcomes in multiple diseases including STS and hepatocellular carcinoma [15,17]. In fact, some literature suggests pSTAT1 acts as a tumour suppressor while uSTAT1 functions as a tumour promoter [17]. The impact of uSTAT1 is distinct from pSTAT1 and this is likely related to the formation of protein complexes that do not require the Y701 phosphorylation versus those that do.

Considering *STAT1* was co-expressed with *PD-L1* in a cohort of UPS cases that had improved clinical outcomes [5], we investigated the role of STAT1 expression and Y701 phosphorylation in human primary UPS cell lines. We hypothesized that increased STAT1 expression, including the Y701 residue being available for phosphorylation, would decrease malignant properties of UPS cells. We derived STAT1 knockout (STAT1^KO^) primary UPS cell lines and examined the effect of IFN-γ in the STAT1^KO^ cell lines. Further, we leveraged the STAT1^KO^ models to create and characterize primary UPS cell lines that overexpressed wild type or phospho-mutant STAT1 (tyrosine 701 to phenylalanine, Y701F). We observed that the Y701 residue was critical in mediating the majority of STAT1’s antitumour functions although certain transcriptional responses were mediated by unphosphorylated STAT1 that may have antitumour functions in some contexts. This work sheds light on the distinct roles of STAT1 Y701 phosphorylation in malignant cell lines and enhances our understanding of IFN-γ signaling in UPS cells.

## Results and discussion

### The loss of STAT1 increased cell count and decreased IFN-γ-induced cell death and cytokine secretion

To study how the loss of STAT1 expression effects UPS cells, STAT1 knockout (STAT1^KO^) cell lines were generated. A plasmid-free CRISPR-Cas9 approach was applied to five different primary UPS cell lines [18], however, clonal populations with disrupted STAT1 protein expression only persisted from two cases, IHRT4606 and IHRT6665 (S1 Fig).

STAT1, through IFN-γ signaling, is critical in mediating cellular responses beyond immune-mediated tumour killing. One example is STAT1-dependent autonomous cell death in some, but not all systems, which can influence tumor growth dynamics [19–21]. To determine whether STAT1 regulates cell growth in UPS cell lines cell count over 96 hours was assessed in the presence and absence of IFN-γ stimulation. Data from primary cell lines derived from IHRT4606 suggested that regardless of IFN-γ stimulation the STAT1^KO^ cell line had an increased cell count at 96 hours relative to both scramble control cell lines (Fig 1A). In contrast to IHRT4606, IHRT6665 cell lines demonstrated no difference in cell counts at 96 hours between the controls and STAT1^KO^ line in the absence of IFN-γ (Fig 1C). However, when treated with IFN-γ the STAT1^KO^ cell line from IHRT6665 was observed to have an increased cell count at 96 hours. Lastly, in both cases there was no difference in cell count from the STAT1^KO^ cell lines when treated with IFN-γ at 96 hours, suggesting STAT1 is the key mediator of IFN-γ-induced decrease in cell count. This supports a role for STAT1 expression in negatively regulating cell count in UPS although this may be IFN-γ-dependent in the case of IHRT6665.

**Fig 1:**
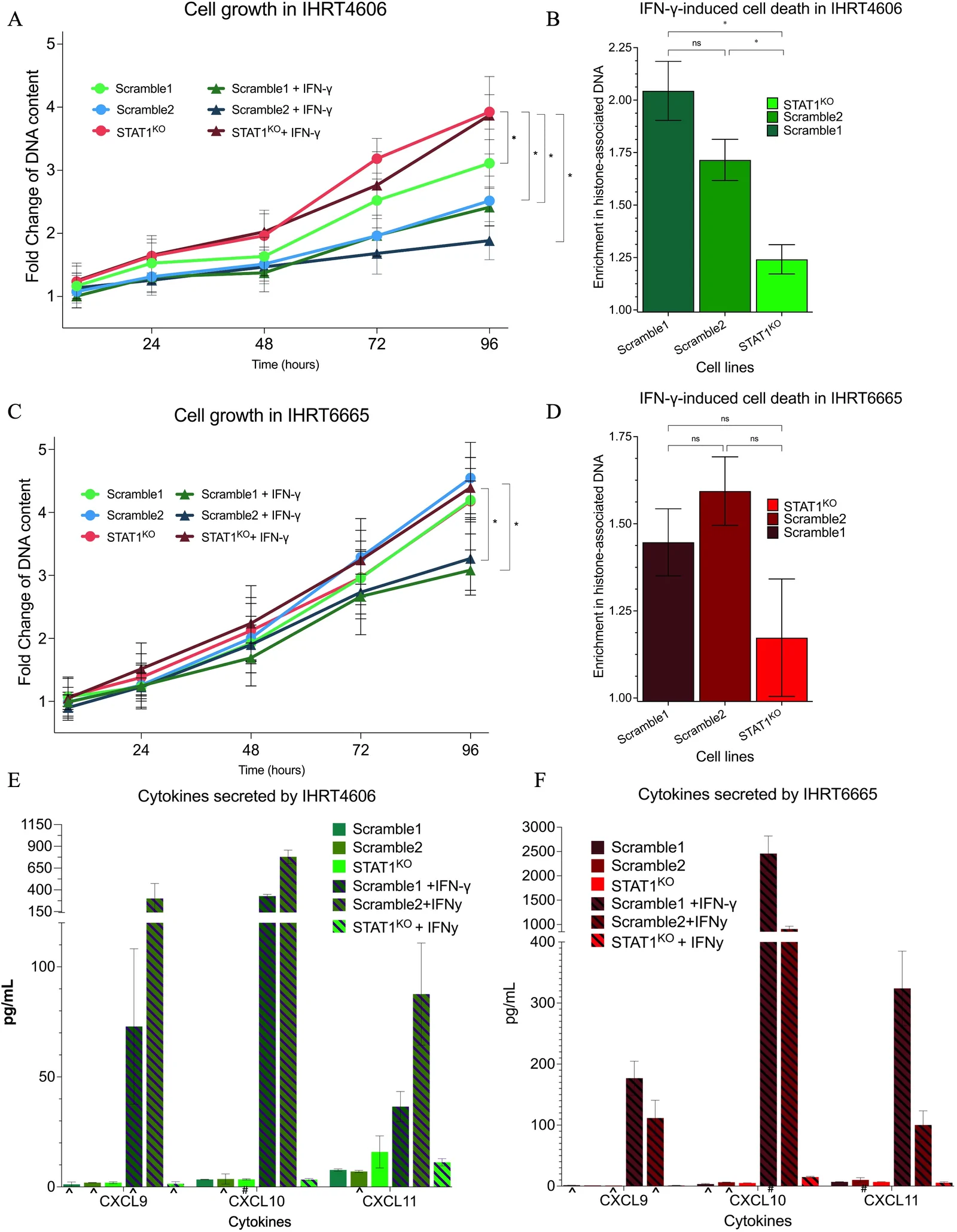
The effect of STAT1 loss on cell growth dynamics and cytokine secretion in UPS cell lines. Cell count and IFN-γ-induced cell death data are presented for STAT1^KO^ cell lines derived from IHRT4606 in A) and B) respectively, and for IHRT6665 in are presented in C) and D) respectively. Cytokine secretion data are presented for case IHRT4606 in E) and for IHRT6665 in F). * indicates p < 0.05 and ns indicates not significant. Enrichment in cell death is displayed as the proportion of cell death relative to the control value. In cytokine secretion plots the mean concentrations of cytokines were plotted in pg/mL for E) IHRT4606 and F) IHRT6665. ^ Indicates one of the replicates was out of the range of detection (below) while # indicates one of the replicates was an outlier of 10x or more and was excluded.

A decrease in cell count could be related to an increase in cell death or a decrease in cell proliferation thus we assessed cell death by evaluating the relative increase in cytoplasmic histone-associated DNA fragments after cells were treated with IFN-γ for 96 hours. We observed less relative cell death in the STAT1^KO^ lines in comparison to the scramble control lines in both cases (Figs 1B and 1D); however, this was only statistically significant in IHRT4606 (Fig 1B). This was consistent with both STAT1^KO^ cell lines demonstrating increased cell counts after 96 hours of IFN-γ treatment and suggests the observed increase in cell count was related to decreased cell death when STAT1 was absent.

Given that STAT1 can mediate the secretion of CXCR3 ligands in response to IFN-γ, we next determined whether the loss of STAT1 would result in decreased secretion of CXCR3 ligands such as CXCL9, CXCL10 and CXCL11. Indeed, the STAT1^KO^ cell line derived from IHRT4606 was observed to secrete less CXCR3 ligands in the presence of IFN-γ than the two scramble control cell lines (Fig 1E). In the case of IHRT6665 the loss of STAT1 also resulted in the reduced secretion of CXCR3 ligands in the presence of IFN-γ relative to scramble controls (Fig 1F). Taken together, this indicated that the loss of STAT1 negatively impacted the secretion of CXCR3 ligands in both UPS cell lines and indicated that IFN-γ signaling may rely on STAT1 to increase the secretion of CXCR3 ligands.

### The effect of STAT1 loss on the transcriptome of UPS cell lines

STAT1 has been reported to be the primary transcription factor activated by IFN-γ. To confirm that the expression programs mediated by IFN-γ would indeed be disrupted by the loss of STAT1 in primary UPS cell lines, STAT1^KO^ cell lines were treated with IFN-γ before RNA was extracted and sequenced. As expected, no genes were significantly upregulated when STAT1^KO^ cell lines from either case were treated with IFN-γ. However, there were some targets that were significantly downregulated in the presence of IFN-γ only in IHRT4606 (S2 Fig). Pathway and gene ontology analyses on these downregulated genes did not detect any pathways that were significantly enriched. These data support STAT1 as a key effector in the transduction of IFN-γ signaling in UPS cell lines, as the loss of STAT1 did not result in detection of any genes differentially expressed in IHRT6665 and only a few differentially expressed in IHRT4606.

We next investigated which targets were regulated by STAT1 in the absence of IFN-γ signaling in both cell lines by comparing STAT1^KO^ with scramble control cell lines. Overall, 268 genes were commonly differentially expressed across both cases, while 1033 and 417 hits were uniquely differentially expressed in IHRT4606 and IHRT6665, respectively. Of these 268 shared genes, ten were upregulated in both cases, thirteen were upregulated in one case and downregulated in the other, and the remaining 245 were downregulated in both of the STAT1^KO^ cell lines. This suggests certain genes were commonly upregulated while other targets were uniquely differentially expressed. Ingenuity Pathway analyses were conducted to identify pathways impacted by the loss of STAT1 (Fig 2). Many pathways were negatively enriched in both cases whereas RHOGDI signaling (Rho GDP dissociation inhibitor) was the only positively enriched pathway in IHRT6665. Some of the top negatively enriched pathways included generic transcription, MHC class I signaling, as well as IFN-α/β signaling, and cell cycle checkpoints. The negative enrichment in generic transcription was supported by decreased expression of multiple zinc finger proteins, which could impair transcription of gene expression programs through decreased or dysregulated transcription factor activity. In addition, this is consistent with STAT1’s role as a transcriptional activator given that many genes differentially expressed and common to both cases were down regulated in the absence of STAT1.

**Fig 2:**
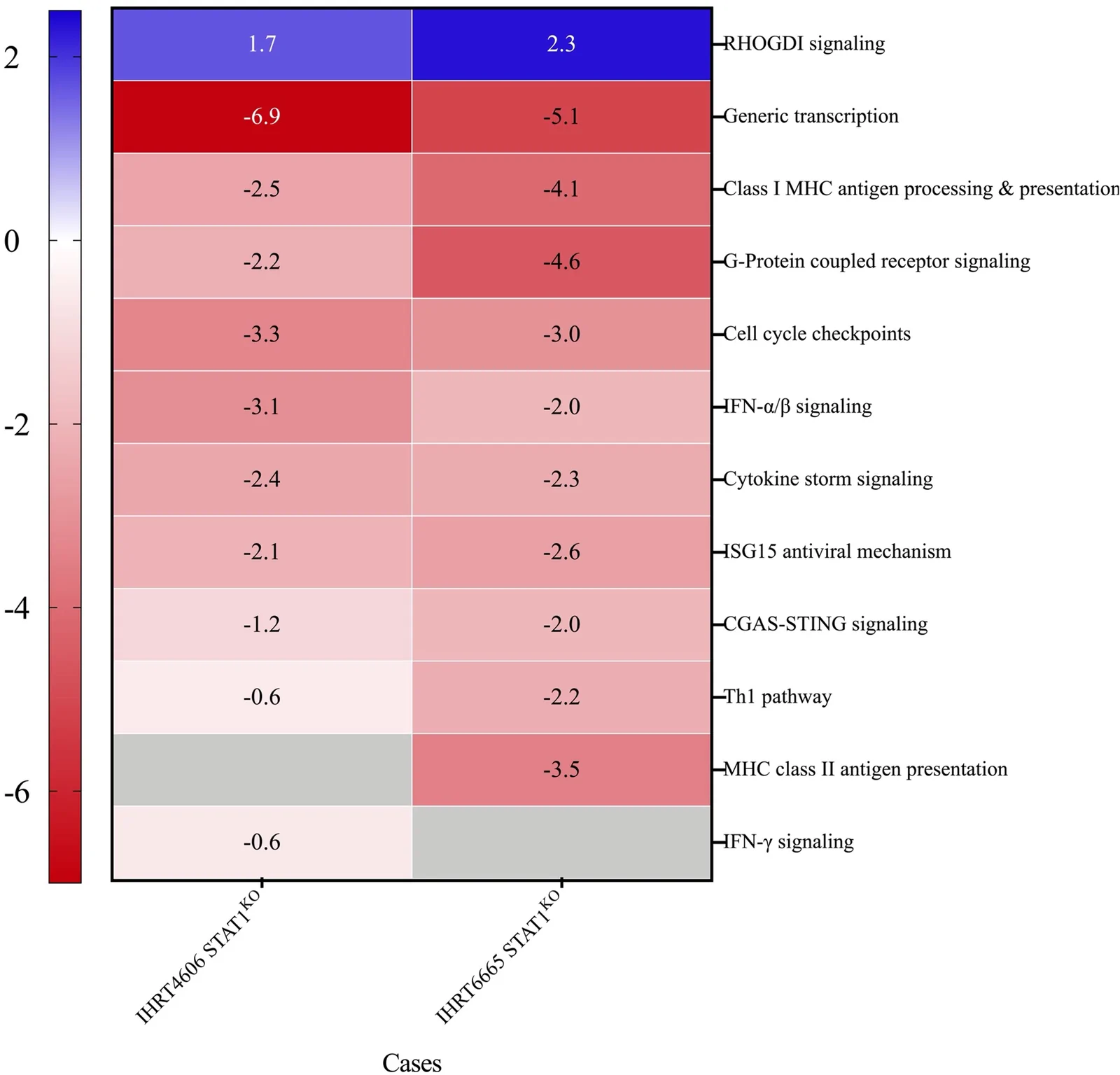
A heatmap of Z scores for pathways that were enriched with genes differentially expressed when STAT1 was knocked out. A Z-score of greater than |2| is considered a significant effect size.

MHC class I signaling is mediated by STAT1, and the negative enrichment of this pathway in the absence of STAT1 has the potential to contribute to tumour-immune evasion. The MHC class II pathway is more commonly active in antigen presenting cells such as macrophages and dendritic cells; however, some tumour cells can express MHC class II molecules. Moreover, MHC class II molecules have been observed to have bimodal or tripart expression patterns in some *in vitro* cancer models such as in colorectal organoids and melanoma cell lines including a subset of models that did not upregulate MHC class II signaling in response to IFN-γ [22–24]. It is possible that some cell lines, such as IHRT4606, may also demonstrate distinct patterns of MHC class II gene expression (Fig 2).

Genes related to cell cycle checkpoints were negatively enriched in STAT1^KO^ cells (Fig 2). This pathway was supported by decreased expression of genes that play a role in sensing and responding to DNA damage. This could suggest cells with unresolved DNA damage pass through the cell cycle if cell cycle checkpoint genes were downregulated. STAT1 can regulate both cell cycle dynamics and cell death through the expression of CDK inhibitors to inhibit the cell cycle [19,25], as well as through the upregulation pro-apoptotic proteins. The observations from this study are consistent with the reported functions of STAT1 in regulating cell cycle progression and cell death.

Lastly and in contrast to IFN-α/β signaling, IFN-γ signaling was not significantly negatively enriched in genes differentially expressed by STAT1^KO^ cell lines. This could suggest that without IFN-γ activation via exogenous treatment, basal IFN-γ signaling in scramble control cell lines was minimal. Although gene sets regulated by IFN-α/β and IFN-γ have some overlap, they are driven by different STAT1 containing protein complexes. Specifically, the Y701 phosphorylation of STAT1 is reported to mediate whether STAT1 interacts with other phosphorylated or unphosphorylated STATs.

### The effect of STAT1 Y701 phosphorylation on cell count, cell death and cytokine secretion in UPS cell lines

To further study how STAT1 expression and IFN-γ signaling impact UPS cell behavior we investigated the effect of the Y701 phosphorylation of STAT1 on UPS cell properties. Two STAT1^KO^ cell lines were transfected with plasmids containing CMV-driven wild type STAT1 (pSTAT1), Y701F phospho-mutant STAT1 (uSTAT1) or an empty vector control (EV). Protein expression of pSTAT1 and total STAT1 in the cell lines was assessed over 96 hours in the presence and absence of IFN-γ to confirm successful cell line derivation. As expected, unphosphorylated Y701 STAT1 was expressed by cells containing the Y701F plasmid, while phosphorylated Y701 STAT1 was only expressed in cell lines transfected with wild type STAT1 (S3 Fig). STAT1^KO+WT^, STAT1^KO+Y701F^ and STAT1^KO+EV^ correspond to cell lines in which wild type STAT1, a tyrosine to phenylalanine phospho-mutant STAT1 and the empty vector control, respectively, were transfected into STAT1^KO^ backgrounds.

In the literature uSTAT1 was associated with antiviral activities while pSTAT1 was associated with antiproliferative effects. To investigate whether these associations would be observed in primary human UPS cell lines, cell count was assessed by means of DNA content over 96 hours in the presence and absence of IFN-γ.

Without IFN-γ, the IHRT4606 STAT1^KO+EV^ cell line had an increased cell count at 96 hours relative to the other two cell lines IHRT4606 STAT1^KO+WT^ and IHRT4606 STAT1^KO+Y701F^ (Fig 3A). This was consistent with the findings of the STAT1^KO^ studies presented in Figure 1. In the presence of IFN-γ, only the comparison between the cell counts from the STAT1^KO+EV^ and the STAT1^KO+WT^ cell lines were statistically significant (Fig 3A). This suggested that the increased cell counts of STAT1^KO+EV^ were related to a lack of STAT1; and this effect was not dependent on IFN-γ in the case of IHRT4606.

**Fig 3:**
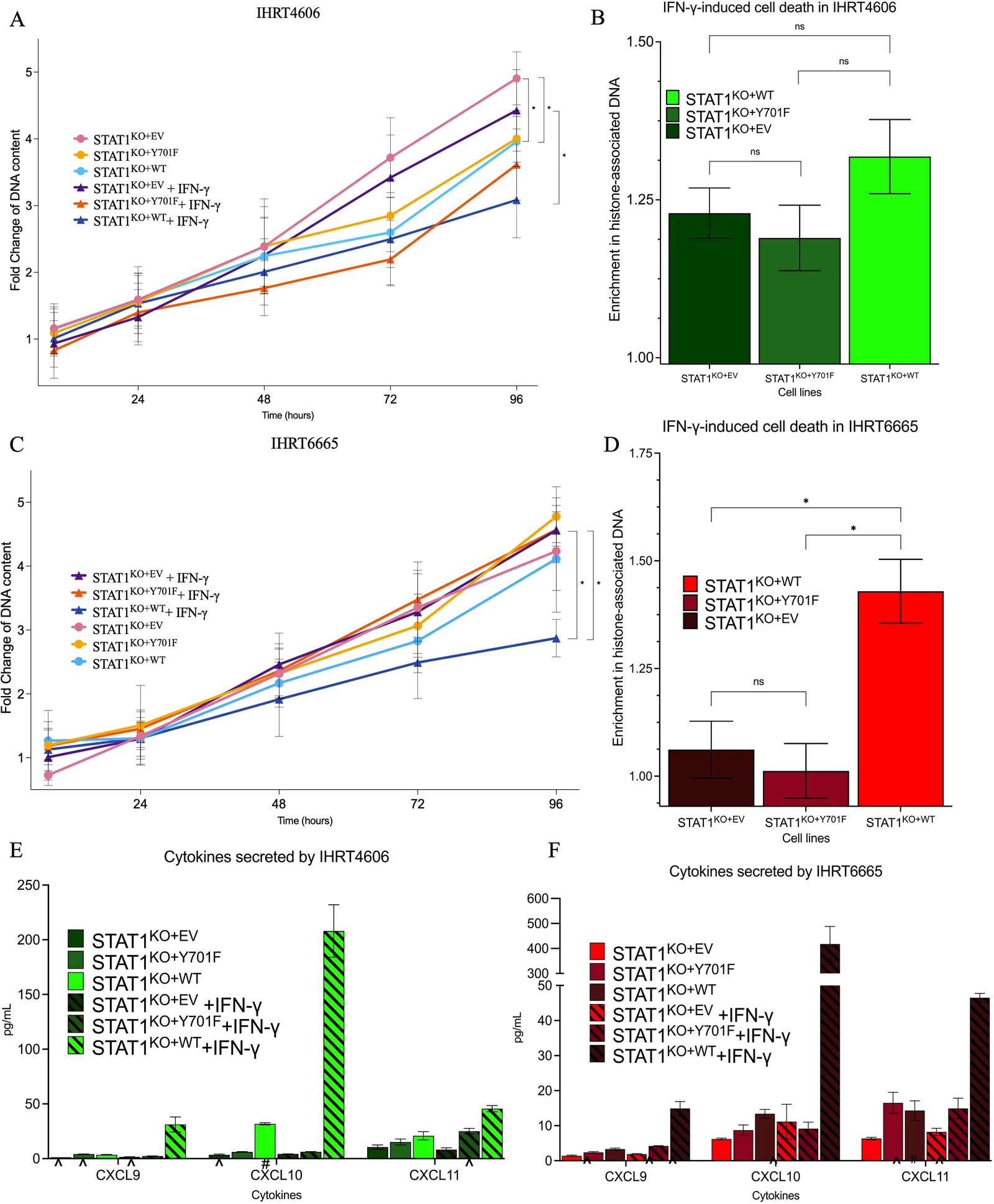
The effect of the STAT1 Y701 phosphorylation on cell growth dynamics and cytokine secretion in UPS cell lines. Cell count and IFN-γ induced cell death for STAT1^KO+EV^, STAT1^KO+WT^ and STAT1^KO+Y701F^ cell lines derived from case IHRT4606 are presented in A) and B) and data from IHRT6665 are presented in C) and D). CXCR3 ligand secretion data are presented for STAT1^KO+EV^, STAT1^KO+WT^ and STAT1^KO+Y701F^ cell lines derived from E) IHRT4606 and F) IHRT6665. * indicated p<0.05 and ns indicates not significant. Enrichment in cell death is displayed as the proportion of cell death relative to the control value. In cytokine secretion plots the mean concentrations of cytokines were plotted in pg/mL. ^ indicates one of the replicates was out of the range of detection (below) while # indicates one of the replicates was an outlier of 10x or more and was excluded.

In the case of IHRT6665, there was no difference in cell count at 96 hours when comparing the STAT1^KO+WT^ or the STAT1^KO+Y701F^ cell lines to the STAT1^KO+EV^ (Fig 3C). In the presence of IFN-γ, the cell count of the STAT1^KO+WT^ cell line was observed to decrease at 96 hours relative to both the STAT1^KO+EV^ and STAT1^KO+Y701F^ cell lines (Fig 3C). This suggested that pSTAT1 was required to facilitate the IFN-γ-mediated decrease in cell count in IHRT6665 and that this effect was eliminated when the Y701 residue was not phosphorylated.

To determine whether the IFN-γ-mediated decrease in cell counts of STAT1^KO+WT^ cell lines was related to a decrease in proliferation or an increase in cell death we examined cell death. It was observed that the STAT1^KO+WT^ cell lines showed the highest amount of IFN-γ-mediated cell death (Figs 3B and 3D), although this was only statistically significant in IHRT6665 (Fig 3D). This demonstrated that the Y701 phosphorylation was related to increased IFN-γ-induced cell death, at least in IHRT6665. The increased cell counts of the STAT1^KO+EV^ and STAT1^KO+Y701F^ cell lines in IHRT6665 in the presence of IFN-γ could be influenced by the decreased amount of relative cell death.

Tumour-secreted cytokines have the potential to shape the microenvironment, and we thus investigated which cytokines were secreted by STAT1^KO+WT^ and STAT1^KO+Y701F^ cell lines. We focused on CXCR3 ligands CXCL9, CXCL10 and CXCL11 as an IFN-γ-mediated increase in these cytokines was limited in the absence of STAT1 (Fig 1E and 1F). To assess this, cell lines were incubated in serum-free media for 24 hours with or without IFN-γ and cytokine concentration was assayed.

CXCR3 ligands were observed to be most highly secreted when STAT1^KO+WT^ cells were treated with IFN-γ (Figs 3E and 3F). Interestingly, CXCL10 was highly secreted by the STAT1^KO+WT^ cell line when treated with IFN-γ in both cases (Figs 3E and 3F). This was consistent with previous observations where CXCL10 secretion was also highly secreted in the presence of IFN-γ (Fig 1E and 1F). This demonstrated a role for phosphorylated STAT1 in the increased secretion of CXCR3 ligands in response to IFN-γ in both cases. As increased CXCL9 and CXCL10 have prognostic and therapeutic implications [26,27], this further supported the idea that the pSTAT1, and not uSTAT1, is important in supporting an anti-tumour microenvironment through IFN-γ signalling.

### The effect of the STAT1 Y701 phosphorylation on transcriptomic profile of UPS cells

Considering STAT1 is a transcription factor and antitumour roles for STAT1 have been associated with the phosphorylation of the Y701 residue, transcriptional profiles of UPS cell lines expressing uSTAT1 and pSTAT1 were investigated. RNA samples from STAT1^KO+EV^, STAT1^KO+WT^ and STAT1^KO+Y701F^ cell lines were subjected to RNA sequencing. These samples were not treated with IFN-γ. Differential expression analyses were run on the transcriptomic profiles to identify targets upregulated in STAT1^KO+Y701F^ cell lines in comparison to STAT1^KO+WT^ cell lines (p-adj < 0.05 and an absolute Log2 fold change >1). Based on the literature in other cell types, we expected to identify genes reported to be regulated by uSTAT1, such as *LMP2*, to be upregulated in STAT1^KO+Y701F^ cell lines [11,14].

Over 700 genes were differentially expressed between STAT1^KO+WT^ and STAT1^KO+Y701F^ in IHRT4606 cells, whereas in IHRT6665 there were 34 differentially expressed genes, 13 of which were shared by both cell lines (Table 1). Interestingly, many of the genes commonly downregulated in STAT1^KO+Y701F^ cell lines were interferon stimulated genes (ISGs) related to antiviral functions. For example, *IRF9* was observed to be commonly downregulated in STAT1^KO+Y701F^ cell lines from both cases. This was expected considering that IRF9 has been shown to only bind to pSTAT1, and not uSTAT1, and is upregulated as a part of the expression programs that are mediated by pSTAT1 [28,29].

**Table 1:** Genes commonly differentially expressed between STAT1^KO+Y701F^ and STAT1^KO+WT^ cell lines.

Gene Symbol	Gene Name	Associated function(s)	Log2 FC in STAT1^KO+Y701F^ cell lines	
			IHRT4606	IHRT6665
*BST2*	Bone marrow stromal cell antigen 2	Antiviral, type II transmembrane glycoprotein, demonstrated STAT1-dependent increased expression in response to IFN-y	−1.88	−1.51
*GBP4*	Guanylate-binding protein 4	Antiviral, negative regulator of virus-triggered type I interferon production	−2.89	−1.96
*HELZ2*	Helicase with zinc finger 2	Antiviral, nuclear transcriptional co-activator	−1.23	−1.94
*IFI27*	Interferon alpha-inducible protein 27	Antiviral, can promote apoptosis	−1.87	−3.00
*IFI44L*	Interferon-induced protein 44-like	Antiviral, can directly bind to STAT1	−1.06	−3.37
*IFITM1*	Interferon-induced transmembrane protein 1	Antiviral, regulates cell growth	−2.21	−2.43
*IRF9*	Interferon regulatory factor 9	Antiviral, mediates type 1 IFN signaling	−1.90	−2.50
*MX1*	Myxovirus resistance 1	Antiviral, GTPase	−1.46	−2.93
*MX2*	Myxovirus resistance 2	Antiviral, GTPase	−1.52	−2.78
*OAS2*	2’-5’-oligoadenylate synthetase 2	Antiviral, can activate viral RNA degradation	−2.44	−1.69
*PRG4*	Proteoglycan 4	Encodes for a large proteoglycan, important in synovial fluid, can regulate malignant cell proliferation, migration and invasion	2.18	1.43
*SLC15A3*	Solute carrier family 15 member 3	Antiviral, involved in dipeptide import across plasma membrane	−1.15	−1.07
*TMEM140*	Transmembrane protein 140	Adhesion, may regulate cell migration, invasion and proliferation	−1.52	−1.42

FC: Fold change

To further investigate the relevance of these 13 genes we investigated if their expression levels were correlated with *STAT1* expression in two larger UPS cohorts. We compiled a cohort of 24 UPS specimens with bulk RNA sequencing data available (Toronto cohort) and observed that *STAT1* expression levels were correlated with 11 of the 13 genes from Table 1 (S4A Fig). Although both Helicase with Zinc Finger 2 (*HELZ2*) and Interferon Induced Transmembrane Protein 1 (*IFITM1*) encode for interferon-inducible proteins, expression of neither were significantly correlated with *STAT1*. We also found that expression of all but one (proteoglycan 4, *PRG4*) of the 13 genes were correlated with *STAT1* expression in a cohort of 33 UPS specimens from the TCGA SARC cohort (S4B Fig). In the future it would be important to directly investigate if the phosphorylation status of STAT1 is related to the expression of these 13 genes in a larger cohort of UPS.

Since increased *STAT1* expression has been shown to be associated with positive clinical outcomes, we evaluated expression of the 13 gene signature and clinical outcome in primary UPS cases with available follow up information (n = 19 and n = 24 patients, for the Toronto and SARC cohorts, respectively) but did not observe an association with outcome (S5 Fig, Table S1).

The overexpression of STAT1 or phospho-mutant STAT1 did not impact transcriptional programs uniformly across the two cell lines; however, a handful of targets related to antiviral functions were commonly downregulated in samples containing Y701 phosphomutant-STAT1. To further investigate which pathways were enriched within genes differentially expressed between STAT1^KO+Y701F^ and STAT1^KO+WT^ cell lines we employed pathway analyses. Cell lines from IHRT6665 yielded no significant results. For those from IHRT4606, it was observed that genes differentially expressed by the STAT1^KO+Y701F^ in comparison to the STAT1^KO+WT^ cell line were negatively enriched in multiple pathways (Fig 4A). One expected negative enrichment was in the IFN-γ signalling pathway and this was consistent with the established role of phosphorylated STAT1 in mediating IFN-γ signal transduction. Other negatively enriched pathways included IFN-α/β, cytokine storm, IL-6, and IL-10 signaling in addition to cell cycle checkpoints and senescence-related pathways (Fig 4A). The negative enrichment in cell cycle checkpoint signaling in the STAT1^KO+Y701F^ cell line was also observed in STAT1^KO^ studies (Fig 2). Together, this supported a role for STAT1 in regulating cell cycle while the data from Fig 4 suggest this may be related to the phosphorylation of the Y701 residue.

**Fig 4:**
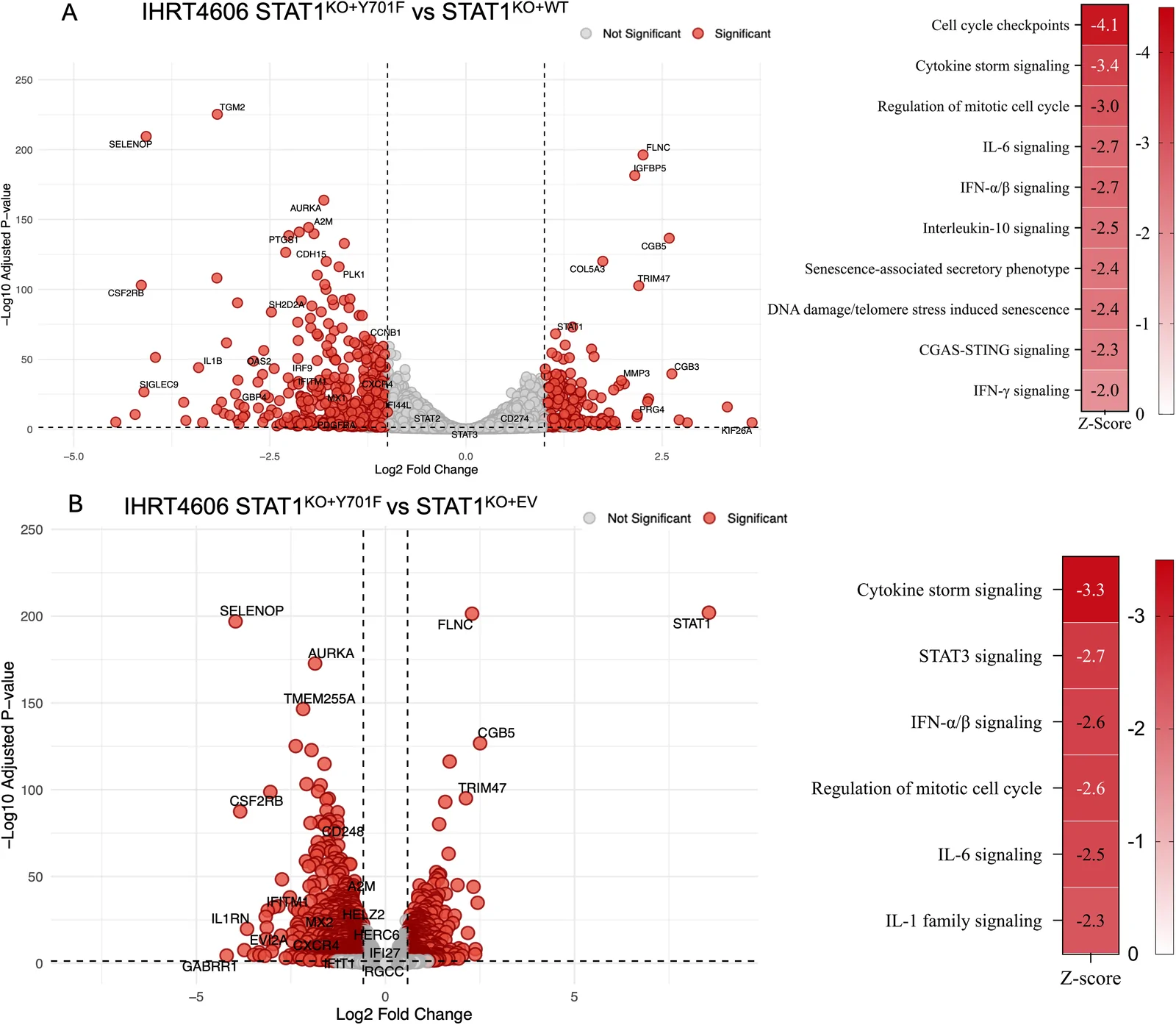
The effect of STAT1 Y701 phosphorylation on the transcriptome of a UPS cell line. Volcano plots of differentially expressed genes and corresponding heatmaps of Z scores for various pathways that were enriched within the genes differentially expressed in comparisons between A) STAT1^KO+Y701F^ vs STAT1^KO+WT^ and B) STAT1^KO+Y701F^ vs STAT1^KO+EV^. A Z-score of greater than |2| is considered a significant effect size.

To understand how the overexpression of uSTAT1 compared to the absence of STAT1 expression we performed a pathway analysis on genes that were differentially expressed between STAT1^KO+Y701F^ and STAT1^KO+EV^ cell lines from IHRT4606 (Fig 4B). Interestingly, we observed overlap between pathways negatively enriched in comparisons between STAT1^KO+Y701F^ and STAT1^KO+EV^ and those negatively enriched between STAT1^KO+Y701F^ and STAT1^KO+WT^ cell lines. This included IFN-α/β, STAT3, cytokine storm, IL-1 family and the IL-6 pathways. These observations could be related to the negative regulatory effects previously reported for uSTAT1. uSTAT1 was observed to negatively regulate the availability of unbound STAT2, STAT3 and other interactors resulting in shifted gene expression programs and protein localization [13,30,31].

Lastly, IFN-γ signaling was not observed to be enriched in genes that were differentially secreted by STAT1^KO+Y701F^ cells in comparison to STAT1^KO+EV^ cells. This supports Y701 phosphorylated STAT1 as a key mediator of IFN-γ signaling even in the absence of IFN-γ stimulation.

## Summary and conclusion

The role of STAT1 expression and Y701 phosphorylation were investigated in UPS, with results summarized in table S2. Overall, the loss of STAT1 expression increased malignant characteristics of UPS cell lines. Without STAT1 cell counts increased, cell death in response to IFN-γ decreased, and the secretion of prominent anti-tumour cytokines decreased. The genes differentially expressed in STAT1^KO^ cell lines were negatively enriched in cell cycle checkpoints as well as many immune-related pathways. Tumour cells that demonstrate decreased IFN-γ-mediated cell death and decreased secretion of CXCR3 ligands may be insensitive to antitumour effects of IFN-γ and this could ultimately implicate tumoral STAT1 expression as a factor in tumour progression.

Given IFN-γ induces STAT1 Y701 phosphorylation, a more granular investigation into how the phosphorylation of this residue would affect UPS cells was undertaken. In the presence of IFN-γ cell counts increased in the STAT1^KO+EV^ cell line in comparison to the STAT1^KO+WT^ cell line. Further, IFN-γ-mediated cell death was highest in STAT1^KO+WT^ cell lines. These observations were consistent with those of the STAT1^KO^ studies. This suggests that the presence of the Y701 phosphorylation was related to the increased IFN-γ mediated cell death, which likely also affected the decreased cell counts that were observed in the IFN-γ-treated STAT1^KO+WT^ cell lines.

Pathway analyses of the genes differentially expressed by the STAT1^KO+Y701F^ versus the STAT1^KO+EV^ cell lines unexpectedly indicated IFN-α/β, IL-6, and STAT3 pathways were negatively enriched. As unphosphorylated STATs can negatively regulate other STATs and transcriptional regulators [30], this could suggest that uSTAT1 is negatively regulating STAT3 and STAT2 by reducing their availability to form other complexes in the STAT1^KO+Y701F^ cell line. On the other hand, pro-tumour signaling pathways such as STAT3 [32–34] signaling were negatively enriched within the STAT1^KO+Y701F^ cell line, indicating that negative regulation of STAT3 signaling by uSTAT1 could have antitumour implications. This suggests that the impact of the Y701 phosphorylation likely has context specific effects.

In conclusion, the Y701 phosphorylation of STAT1 was observed to be important in many anti-tumour gene expression programs in comparison to unphosphorylated STAT1. This could be related to dominant negative effects of uSTAT1 that negatively regulate the activities of other STAT1 binding partners or the inability of STAT1^KO+Y701F^ cells to participate in ISGF3 complex formation and therefore IFN- α/β-associated functions.

## Methods and materials

### Cell culturing and IFN-γ treatment

Primary cell lines were previously established as described below and in detail in [18]. Briefly, fresh or viably cryopreserved tumour fragments from were physically dissociated and incubated in Growth Media (GM) with the addition of 2–10 mg/ml collagenase for 24 hours. GM consisted of Dulbecco’s Modified Eagle Medium/Nutrient Mixture F12 with 1% Penicillin/Streptomycin and 10% Human AB Serum (Cedarlane). When indicated, a concentration of 10ng/mL of IFN-γ (ThermoFisher) was used throughout this study. Media components were procured from ThermoFisher Scientific or Wisent Bioproducts unless otherwise specified. Cultures were then expanded in fresh GM. Cell lines and bulk specimens were molecularly characterized and compared to validate the cell lines. The two cases used in this study were collected from primary adult UPS cases and identified as IHRT4606 and IHRT6665.

The Sinai Health Research Ethics Board (formerly Mount Sinai Hospital (MSH) REB) approval for samples used in this project was first approved on 25, May 2001 and is renewed annually. Recruitment was open on 25, May 2001, and utilized written informed consent.

### Derivation of STAT1 Knockout (KO) primary UPS cell lines

To create STAT1^KO^ cell lines a plasmid-free CRISPR-Cas9 based system was employed. Four crRNAs were used to target *STAT1*, in addition to a positive control crRNA targeting the *HPRT* gene and two negative scramble control crRNAs (IDT, Table S3).

UPS cell lines were transfected with ribonucleoprotein solution of Alt-R™ S.p. HiFi Cas9 Nuclease V3 (IDT), sgRNA and CRISPRMAX transfection reagent (ThermoFisher). Untreated and ‘mock transfection’ conditions were also included. After transfection, approximately 400 UPS cells were plated in six 96 well plates at a density of 0.7 cells/well to expand single cell clonal populations. Genomic DNA from bulk transfected populations were then used to estimate editing efficiency. This was accomplished utilizing Sanger sequencing of PCR amplicons (primer sequences in Table S4) that contained the *STAT1* target exons, and a Tracking of Indels by Decomposition (TIDE, https://tide.nki.nl/) analysis. Expanded clonal populations were then screened for homogeneous edits that disrupted STAT1 protein expression even when cells were treated with IFN-γ for two hours.

### Assessments of cell growth and IFN-γ-induced cell death

To quantify cell growth, assessments of relative increase in DNA content over a period of 96 hours were undertaken. Cell lines were grown overnight in a 96-well plate at a concentration of 2–3.0 x10^3^ cells per well. GM with or without 10ng/ml IFN-γ was added to the wells gently to limit cell detachment. Over 96 hours, the media was aspirated, and the plate was frozen at −20°C. The CyQuant NF cell proliferation assay (ThermoFisher) was used to assess the DNA content of each well, according to the manufacturer’s protocol. The mean fluorescence intensity (MFI) at 8, 24, 48, 72 and 96 hours was compared to the MFI at zero hours. There were five technical replicates in each plate and experiments were performed a minimum of four times. Data were analyzed using an ANOVA (Tukey’s post-hoc) completed in R version 4.3.3.

To assess IFN-γ-induced cell death in cell lines the relative amount of apoptosis-associated DNA fragmentation was quantified. Cytoplasmic histone-associated DNA was quantified using a Cell Death Detection ELISA^PLUS^ kit (Roche) in untreated and IFN-γ-treated cells. Samples were plated in 96 well plates at 2–3.0 x10^3^ cells per well and analyzed after 96 hours of 10ng/mL IFN-γ treatment according to manufacturer’s protocol, where 10μM camptothecin treatment was used as a positive control. Samples were plated in triplicate, and data were averages from a minimum of four independent experiments. IFN-γ-treated samples were compared to untreated samples to determine the enrichment in cytoplasmic histone-associated DNA of the IFN-γ treated samples. For comparisons between scramble guide population 1 and 2 with STAT1^KO^ cells, as well as between pSTAT1 cell lines with either uSTAT1 or EV control cell lines, an ANOVA (Tukey’s post-hoc) in R version 4.3.3. was used.

### Analysis of cytokines secreted by UPS cells

Secreted cytokines from UPS cell lines were evaluated as follows. 7.5x10^4^ cells/well were plated into a 24-well plate and were left to settle for 12–18 hours before being washed with PBS. Serum-free GM with or without 10ng/ml of IFN-γ was then added. After 24 hours the supernatant was collected promptly, cold centrifuged, aliquoted and immediately stored in −80°C.

Supernatant from IFN-γ-treated or untreated cell lines were analyzed using a ProCarta-Plex custom kit (ThermoFisher). Samples were analyzed for CXCL9, CXCL10 and CXCL11 in triplicate using the Luminex platform according to the manufacturer’s protocol. Quantification of secreted cytokines was performed at The Princess Margaret Genomics Centre in Toronto, Ontario. A standard curve method was used to determine the concentration of the secreted cytokines in pg/mL. Data are presented as the pg/mL values with error bars representing + /- SEM.

### Protein expression analysis

To identify the expression of specific proteins, eight to thirty micrograms of total protein from each sample was loaded into the well of a 7.5% SDS-Polyacrylamide gel (BioRad), run, and later transferred to a nitrocellulose membrane (ThermoFisher) at 4°C overnight. After blocking, membranes were incubated with a primary antibody overnight. Primary antibodies and the dilutions utilized were as follows: rabbit anti-pSTAT1 (1:2000, clone 58D6, Cell Signaling) and mouse or rabbit anti-STAT1 (both 1:2500, clone 42, BD Transduction Laboratories and clone 9172, Cell Signaling, respectively). Secondary goat anti-rabbit or donkey anti-mouse antibody conjugated to horseradish peroxidase at a dilution of 1:10000 in 1xTBS-T were used. The loading control for each experiment was a mouse anti ß-actin antibody (clone AC-74, Sigma), utilized at a dilution of 1:3000. Membranes were visualized with SuperSignal™ West Pico PLUS Chemiluminescent Substrate (ThermoFisher) and CL-XPosure™ Film (ThermoFisher).

### RNA sequencing and transcriptomic analyses

Transcriptome sequencing was performed by The Centre for Applied Genomics (TCAG) in Toronto, ON, Canada. Samples were collected from three independent experiments. 100ng of cell line RNA was extracted and libraries were prepared prior to sequencing on Illumina’s NovaSeq 6000. Read pre-processing, alignment, quality control and initial exploratory analyses were performed by TCAG. Statistical significance was considered at P < 0.05. Ingenuity pathway analyses (IPA) were completed using IPA version 01-23-01. The Z-score generated from IPA indicated the effect size of the enrichment where a value greater than |2| is used as a cut off to indicate a significant effect size.

### Statistical analysis

Correlations between STAT1 expression and other genes were assessed using Pearson correlation with Benjamini–Hochberg FDR correction for multiple testing.

A gene signature was derived from genes commonly differentially expressed between wild type and phospho-mutant STAT1 cell lines. Associations between the signature and overall survival were evaluated using Kaplan–Meier survival analysis and univariate Cox proportional hazards regression within two independent cohorts. Briefly, in the Toronto cohort, count data were filtered to remove lowly expressed genes and variance-stabilizing transformed (VST) using the DESeq2 package version 1.42.1 in R version 4.3.3. In the TCGA cohort TPM data were log transformed as log2(TPM + 1). Expression of each signature gene was standardized using gene-wise z-scores and used to calculate a signature score for each case. Cases were stratified into high- and low-signature groups using the median of each cohort. Then, UPS cases with available follow-up information and those from patients who did not present with a prior malignancy were included in the survival analysis (Toronto Cohort n = 19; TCGA n = 24). All analyses, including univariate Cox proportional hazards regressions were performed separately within each cohort (R packages survival, survminer, and ggplot2 versions 3.8−3, 0.5.1 and 3.5.2 respectively). Statistical significance was considered at P < 0.05.

### Derivation of wildtype and phosphomutant STAT1-overexpressing UPS cell lines

To create STAT1 wild type and phospho-mutant overexpression cell lines, UPS lines were edited to disrupt the production of STAT1 protein. HEK239TN cells (6x10^5^ cells per plate, plated 12–18 hours before transfection) were transfected with psPAX2, VSV.g (gifts from Dr. Gingras) and one of three transfer plasmids using Lipofectamine 3000 (ThermoFisher) to create lentiviral particles. The three CMV-driven transfer plasmids included an empty vector control (EV), wild type STAT1, or the phospho-mutant STAT1 (701 Y > F) plasmid (the latter two plasmids were gifts from Dr. George Stark [12],).

Confluent ten cm plates of UPS STAT1^KO^ cell lines were then infected with the lentiviral particles. Cell lines derived here are referred to as STAT1^KO+Y701F^ (CMV-STAT1-Y701F), STAT1^KO+WT^ (CMV-STAT1-WT) and STAT1^KO+EV^ (CMV-EV) along with the IHRT number of the bulk specimen they were derived from.

## Supporting information

S1 Table Overall survival analysis based on signature group. A) The results of a Cox proportional hazard analysis in the Toronto cohort and B) in the TCGA SARC cohort of UPS.
(XLSX)

S2 Table A summary of genetic manipulations and experimental outcomes for cell count, cel death and cytokine secretion. (XLSX)

S3 Table Sequences used to target *STAT1* by gRNAs. (XLSX)

S4 Table Primers used to amplify regions where CRISPR-Cas9-mediated DNA edits were expected. (XLSX)

S5 Table List of abbreviations. (XLSX)

S1 Fig Visualization of protein expression of Y701 phosphorylated STAT1 and total STAT1 in STAT1^KO^ UPS cell lines derived from A) IHRT4606 and B) IHRT6665. STAT1 protein expression was not present in the knockout conditions in the absence and presence of IFN-γ from cell lines derived from.
(PDF)

S2 Fig An assessment of differentially expressed genes in STAT1^KO^ cell lines in the presence of IFN-γ. A) IHRT4606 and B) IHRT6665 STAT1^KO^ cell lines were treated with ten ng/mL IFN-γ for eight hours. The genes that were differentially expressed across the treated and untreated conditions using (adjusted p value of less than 0.05 and absolute Log2 fold change greater than two) as criteria for significantly differentially expressed.
(PDF)

S3 Fig A visualization of protein expression of Y701 phosphorylated and total STAT1 in STAT1^KO+WT^, STAT1^KO+Y701F^ and STAT1^KO+EV^ cell lines over 96 hours with and without IFN-γ treatment. A band corresponding to STAT1 expression was seen all lanes except the lane of the STAT1^KO+EV^ cell lines. Y701 pSTAT1 was expressed in the lanes corresponding to STAT1^KO+WT^ but not in STAT1^KO+EV^ or STAT1^KO+Y701F^ cell lines. β-actin was used as a loading control. A) The top panel depicts the blots from IHRT4606, and B) the lower panel depicts those from IHRT6665.
(PDF)

S4 Fig Pearson correlations between expression of STAT1 and 13 genes from Table 1 in two UPS cohorts. A) The correlation between expression of STAT1 and 13 genes differentially expressed based on STAT1 phosphorylation from A) the Toronto cohort (n = 24) and B) the TCGA SARC cohort of UPS (n = 33).
(PDF)

S5 Fig Kaplan–Meier overall survival (OS) curves based on a 13-gene signature. **Data** from A) the Toronto UPS cohort (n = 19) and B) the TCGA SARC cohort of UPS (n = 24), where follow-up information was available for those who did not present with a prior malignancy. The signature was derived from 13 genes differentially expressed based on STAT1 phosphorylation.
(PDF)

S1 Raw Images Scans of western blot films that correspond to supporting figures. (PDF)
