# Supplementary material for: The effect of STAT1 expression and the Y701 phosphorylation on primary Undifferentiated Pleomorphic Sarcoma cells
Source: PLoS One. 2026 Oct 26;21(10):e0361295. doi: 10.1371/journal.pone.0361295 (PMC13704806; doi:10.1371/journal.pone.0361295)
Supplement: Supplementary file 1 [file pone.0361295.s006.pdf]

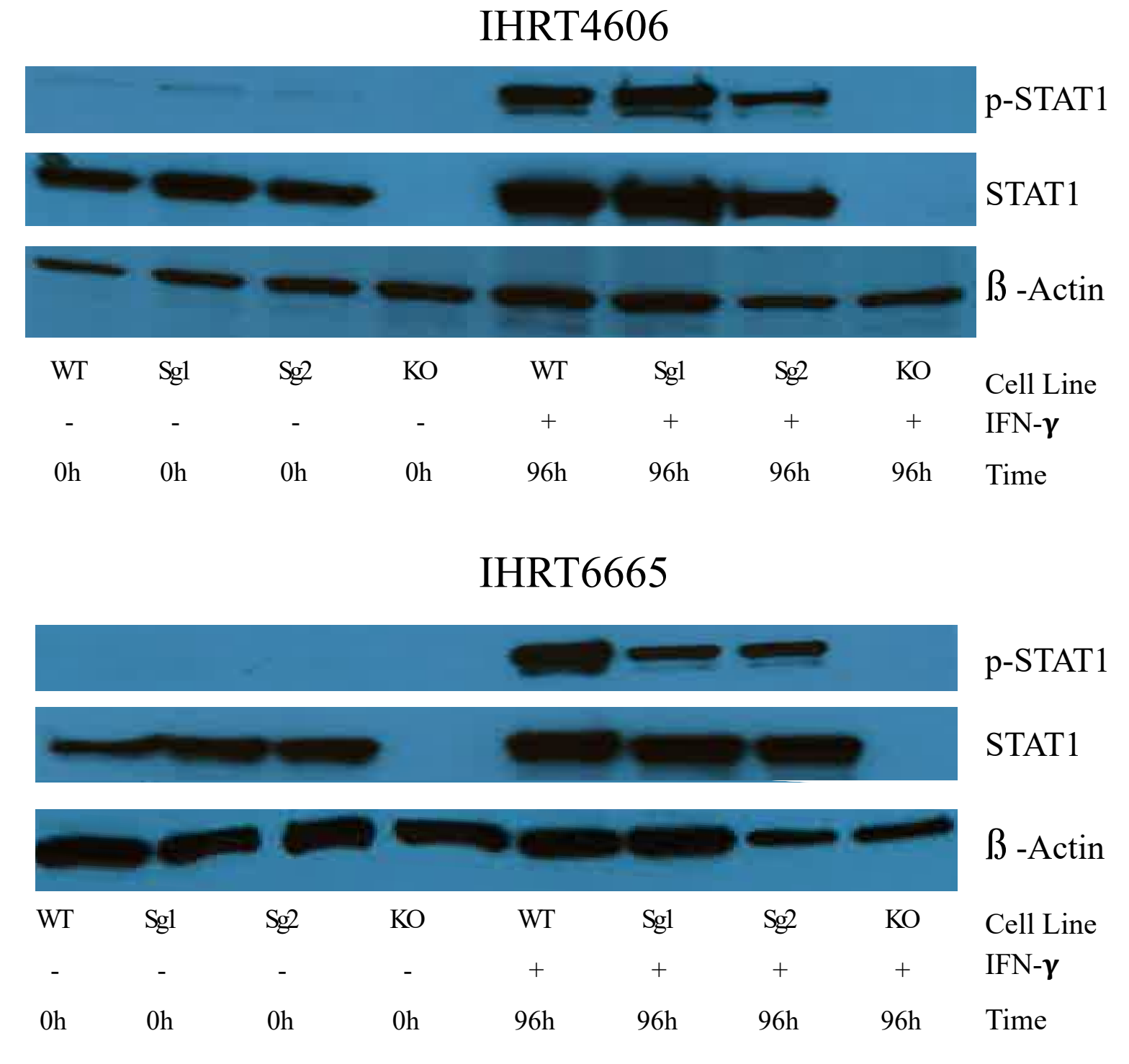
IHRT4606
p-STAT1
STAT1
ß -Actin
WT Sg1 Sg2 KO WT Sg1 Sg2 KO Cell Line
- - - - + + + + IFN-γ
0h 0h 0h 0h 96h 96h 96h 96h Time
IHRT6665
p-STAT1
STAT1
ß -Actin
WT Sg1 Sg2 KO WT Sg1 Sg2 KO Cell Line
- - - - + + + + IFN-γ
0h 0h 0h 0h 96h 96h 96h 96h Time
