# Supplementary material for: The effect of STAT1 expression and the Y701 phosphorylation on primary Undifferentiated Pleomorphic Sarcoma cells
Source: PLoS One. 2026 Oct 26;21(10):e0361295. doi: 10.1371/journal.pone.0361295 (PMC13704806; doi:10.1371/journal.pone.0361295)
Supplement: Supplementary file 2 [file pone.0361295.s007.pdf]

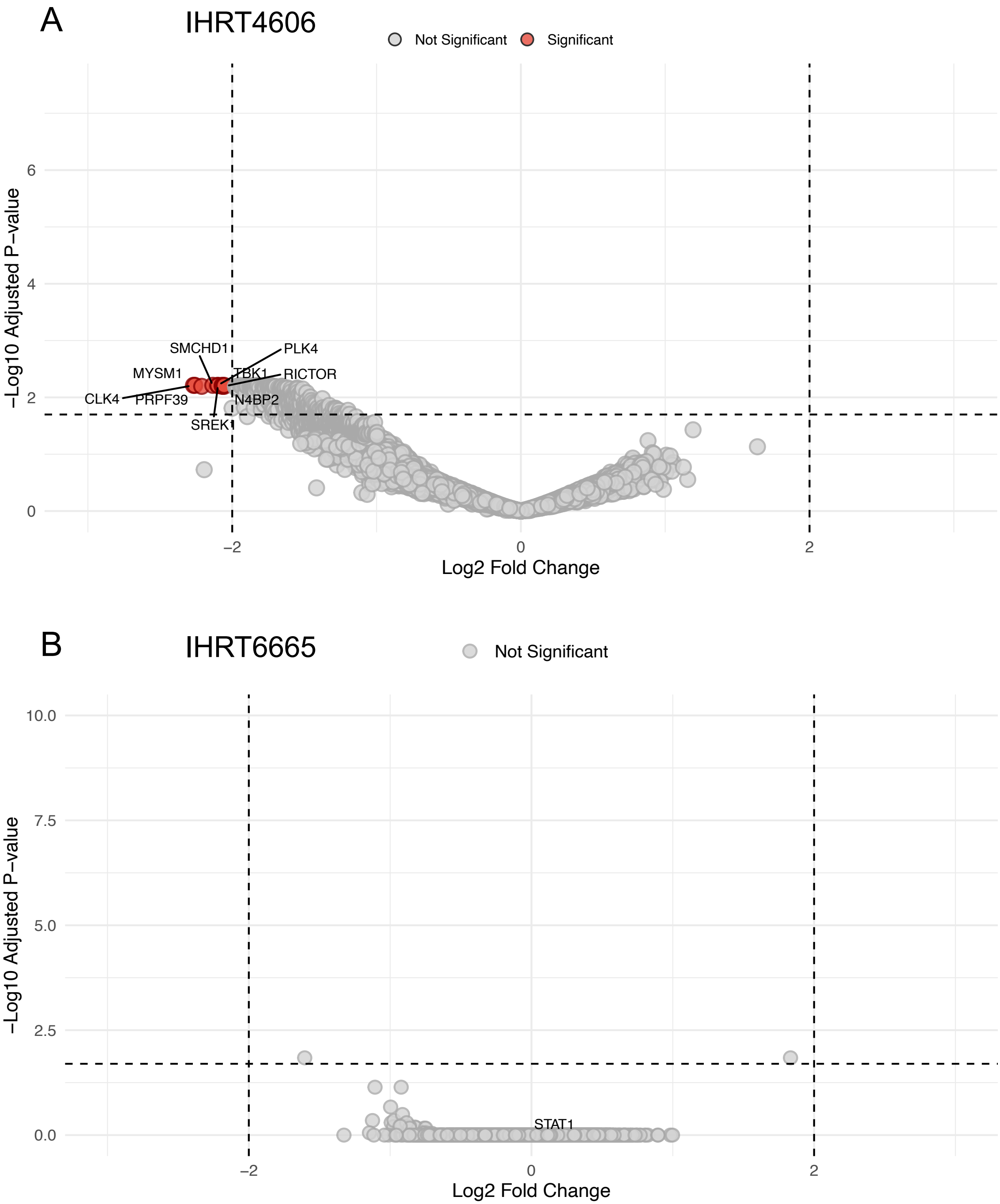
A
IHRT4606
Not Significant
Significant
−Log10 Adjusted P−value
Log2 Fold Change
SMCHD1
PLK4
MYSM1
TBK1
RICTOR
CLK4
PRPF39
N4BP2
SREK1
B
IHRT6665
Not Significant
−Log10 Adjusted P−value
Log2 Fold Change
STAT1
