# Supplementary material for: The effect of STAT1 expression and the Y701 phosphorylation on primary Undifferentiated Pleomorphic Sarcoma cells
Source: PLoS One. 2026 Oct 26;21(10):e0361295. doi: 10.1371/journal.pone.0361295 (PMC13704806; doi:10.1371/journal.pone.0361295)
Supplement: Supplementary file 3 [file pone.0361295.s008.pdf]

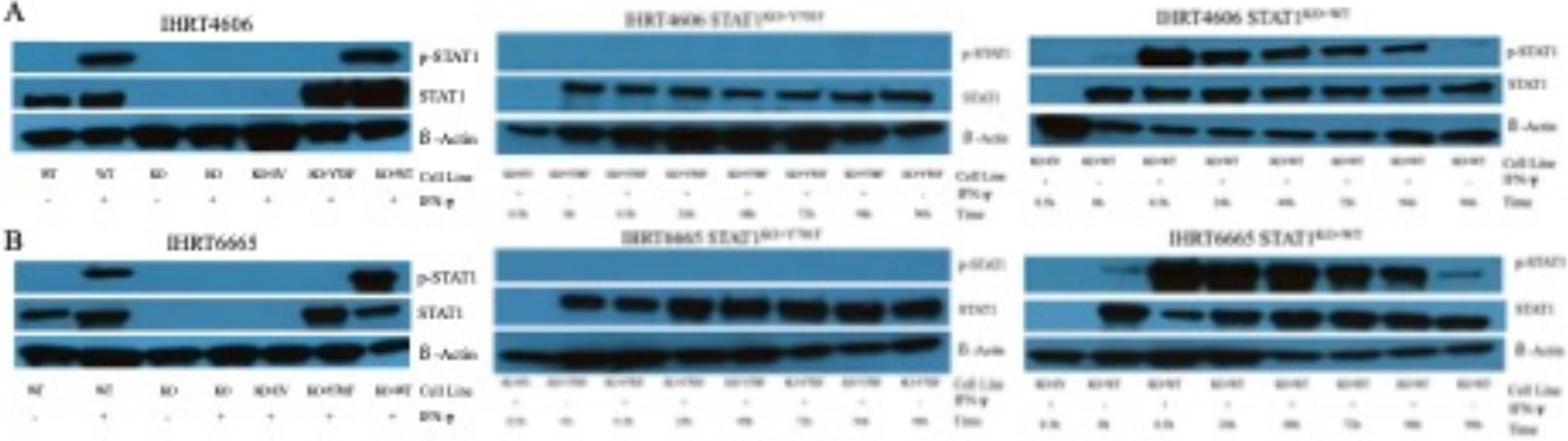
A
IHRT4606
p-STAT1
STAT1
β-Actin
Cell Line
IFN-γ
B
IHRT6665
p-STAT1
STAT1
β-Actin
Cell Line
IFN-γ
