# Supplementary material for: The effect of STAT1 expression and the Y701 phosphorylation on primary Undifferentiated Pleomorphic Sarcoma cells
Source: PLoS One. 2026 Oct 26;21(10):e0361295. doi: 10.1371/journal.pone.0361295 (PMC13704806; doi:10.1371/journal.pone.0361295)
Supplement: Supplementary file 4 [file pone.0361295.s009.pdf]

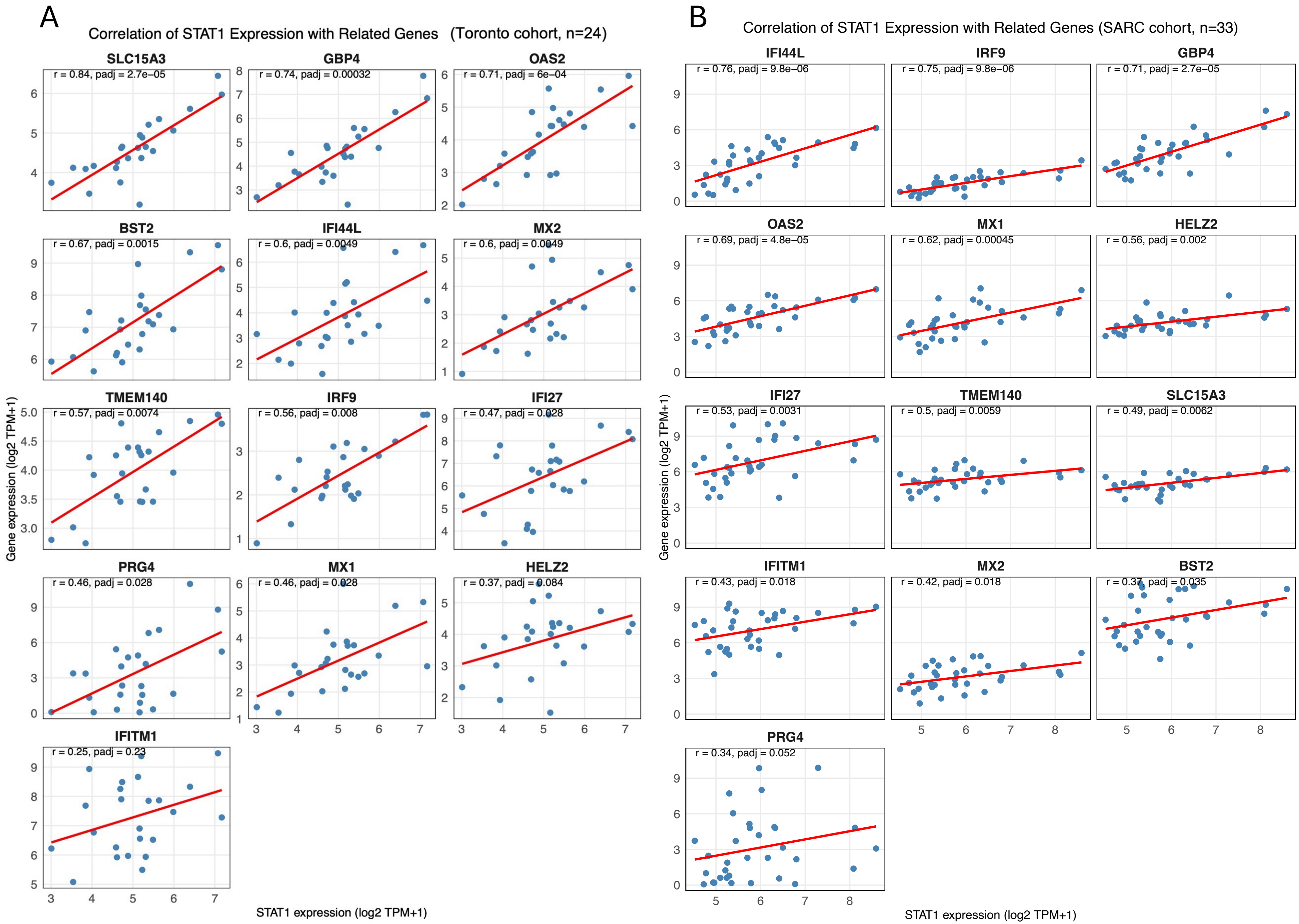
A
Correlation of STAT1 Expression with Related Genes (Toronto cohort, n=24)
SLC15A3
r = 0.84, padj = 2.7e−05
GBP4
r = 0.74, padj = 0.00032
OAS2
r = 0.71, padj = 6e−04
BST2
r = 0.67, padj = 0.0015
IFI44L
r = 0.6, padj = 0.0049
MX2
r = 0.6, padj = 0.0049
TMEM140
r = 0.57, padj = 0.0074
IRF9
r = 0.56, padj = 0.008
IFI27
r = 0.47, padj = 0.028
PRG4
r = 0.46, padj = 0.028
MX1
r = 0.46, padj = 0.028
HELZ2
r = 0.37, padj = 0.084
IFITM1
r = 0.25, padj = 0.23
Gene expression (log2 TPM+1)
STAT1 expression (log2 TPM+1)
B
Correlation of STAT1 Expression with Related Genes (SARC cohort, n=33)
IFI44L
r = 0.76, padj = 9.8e−06
IRF9
r = 0.75, padj = 9.8e−06
GBP4
r = 0.71, padj = 2.7e−05
OAS2
r = 0.69, padj = 4.8e−05
MX1
r = 0.62, padj = 0.00045
HELZ2
r = 0.56, padj = 0.002
IFI27
r = 0.53, padj = 0.0031
TMEM140
r = 0.5, padj = 0.0059
SLC15A3
r = 0.49, padj = 0.0062
IFITM1
r = 0.43, padj = 0.018
MX2
r = 0.42, padj = 0.018
BST2
r = 0.37, padj = 0.035
PRG4
r = 0.34, padj = 0.052
Gene expression (log2 TPM+1)
STAT1 expression (log2 TPM+1)
