# Supplementary material for: The effect of STAT1 expression and the Y701 phosphorylation on primary Undifferentiated Pleomorphic Sarcoma cells
Source: PLoS One. 2026 Oct 26;21(10):e0361295. doi: 10.1371/journal.pone.0361295 (PMC13704806; doi:10.1371/journal.pone.0361295)
Supplement: Supplementary file 5 [file pone.0361295.s010.pdf]

A

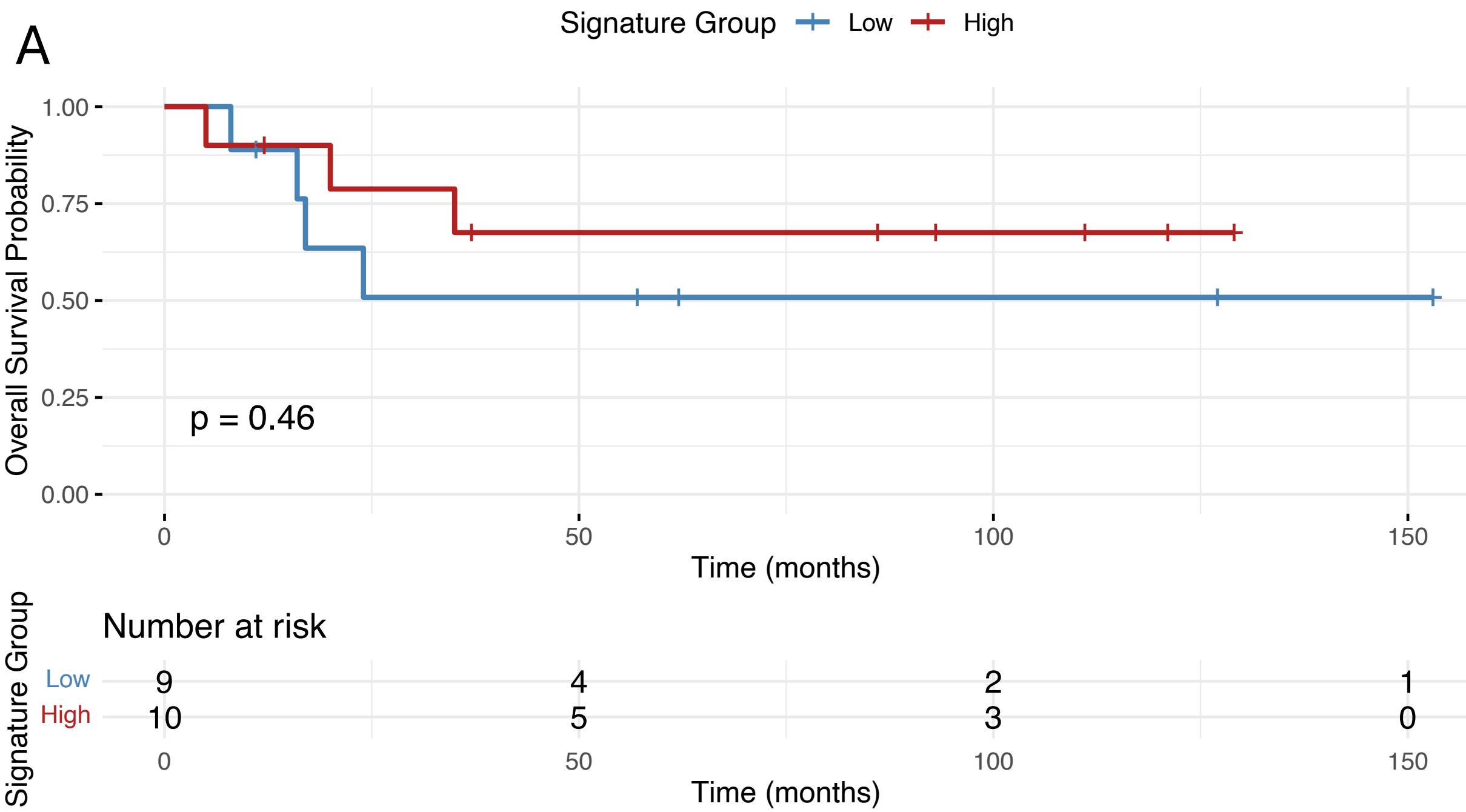
Signature Group
Low
High
Overall Survival Probability
1.00
0.75
0.50
0.25
0.00
p = 0.46
0
50
100
150
Time (months)
Number at risk
Signature Group
Low
High
9
4
2
1
10
5
3
0
0
50
100
150
Time (months)

B

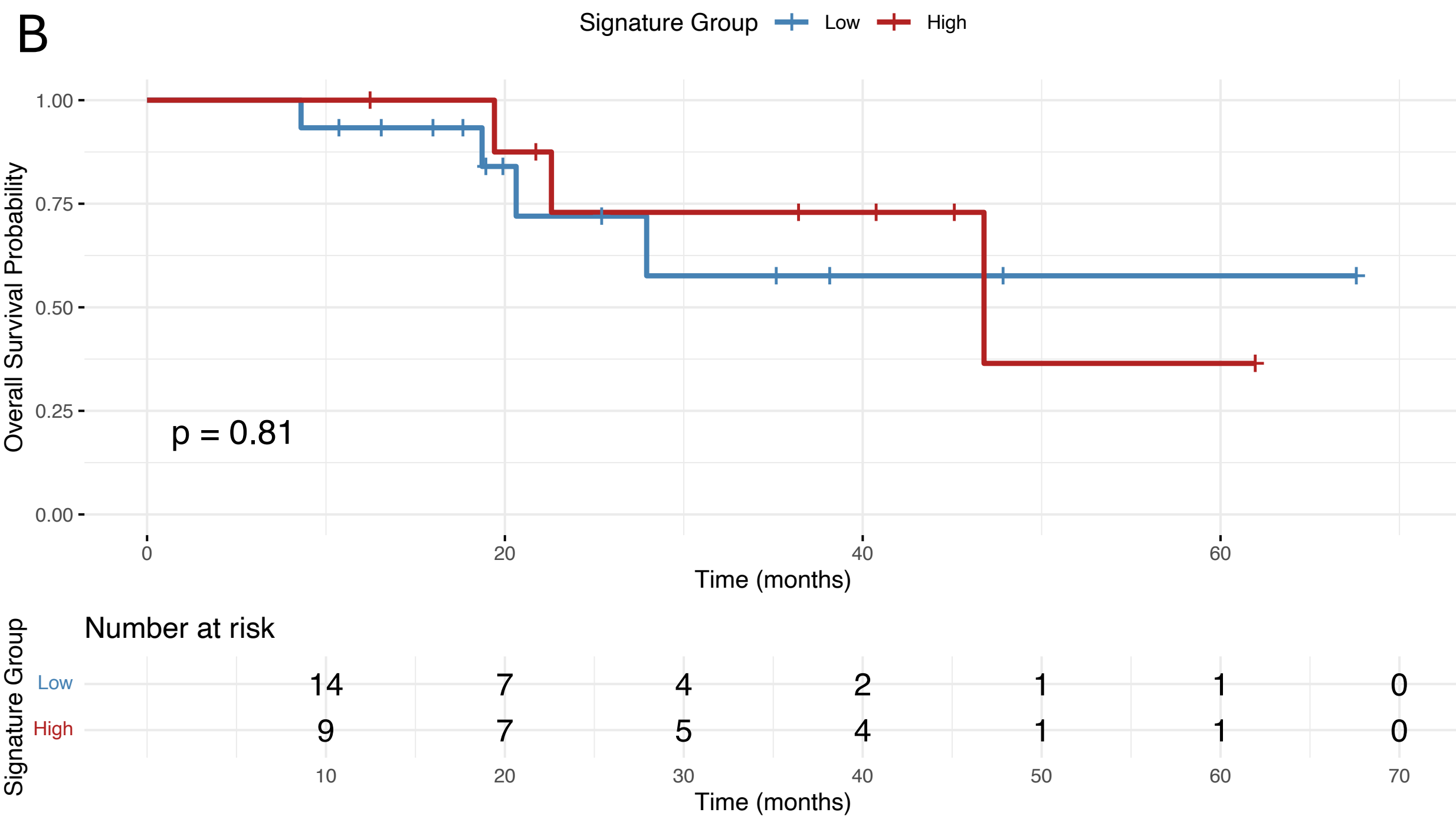
Signature Group
Low
High
Overall Survival Probability
1.00
0.75
0.50
0.25
0.00
p = 0.81
0
20
40
60
Time (months)
Number at risk
Signature Group
Low
High
14
7
4
2
1
1
0
9
7
5
4
1
1
0
10
20
30
40
50
60
70
Time (months)
