# Supplementary material for: The effect of STAT1 expression and the Y701 phosphorylation on primary Undifferentiated Pleomorphic Sarcoma cells
Source: PLoS One. 2026 Oct 26;21(10):e0361295. doi: 10.1371/journal.pone.0361295 (PMC13704806; doi:10.1371/journal.pone.0361295)

IHRT4606 supplementary figure 1

Loading order (Left to right):

1. Ladder
2. WT 0h 0ng/ml IFN-γ
3. Scramble guide 1 0h 0ng/ml IFN-γ
4. Scramble guide 2 0h 0ng/ml IFN-γ
5. STAT1 **knockout** 0h 0ng/ml IFN-γ
6. WT 96h 10ng/ml IFN-γ
7. Scramble guide 1 96h 10ng/ml IFN-γ
8. Scramble guide 2 96h 10ng/ml IFN-γ
9. STAT1 **knockout** 96h 10ng/ml IFN-γ

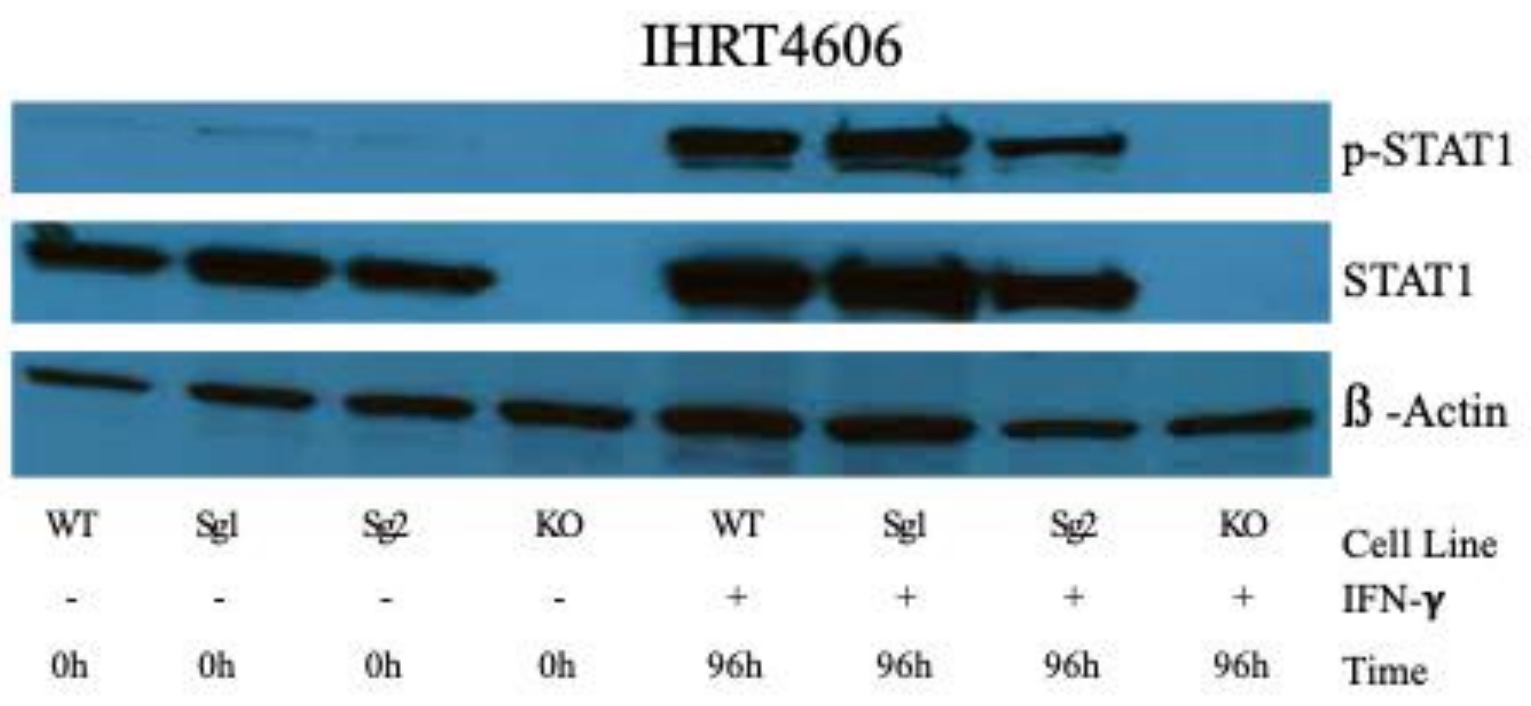

STAT1

pSTAT1

beta actin

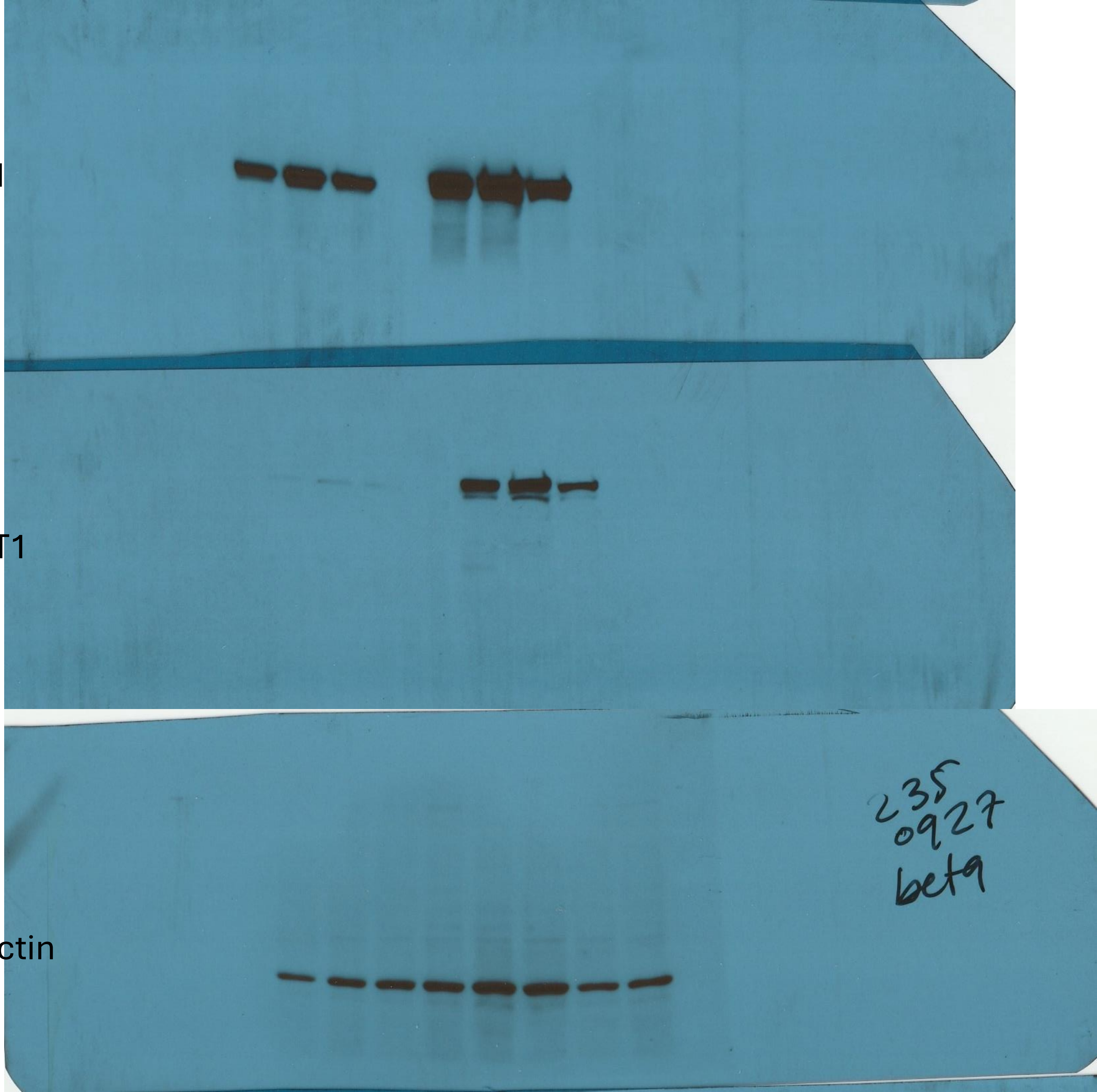

IHRT6665 supplementary figure 1

Loading order (Left to right):

1. Ladder
2. WT 0h 0ng/ml IFN-γ
3. Scramble guide 1 0h 0ng/ml IFN-γ
4. Scramble guide 2 0h 0ng/ml IFN-γ
5. STAT1 **knockout** 0h 0ng/ml IFN-γ
6. WT 96h 10ng/ml IFN-γ
7. Scramble guide 1 96h 10ng/ml IFN-γ
8. Scramble guide 2 96h 10ng/ml IFN-γ
9. STAT1 **knockout** 96h 10ng/ml IFN-γ

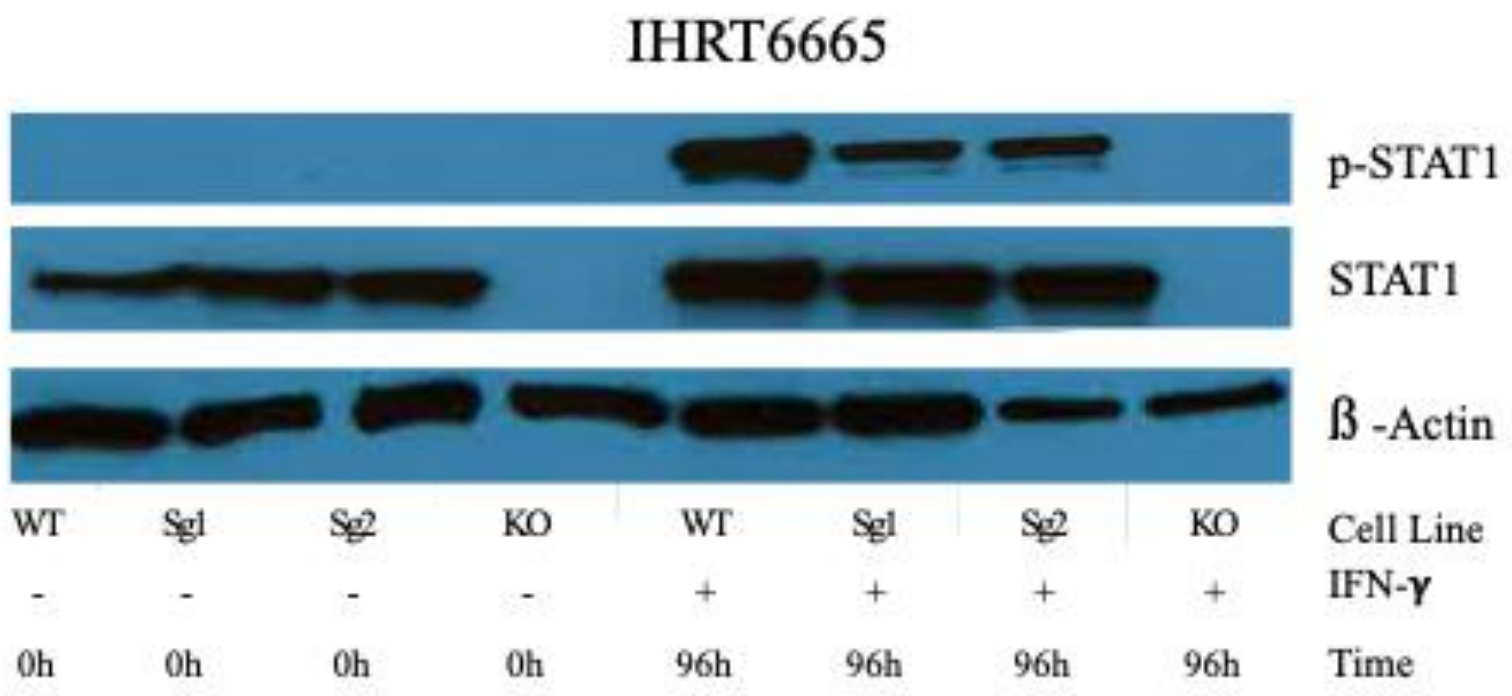

pSTAT1

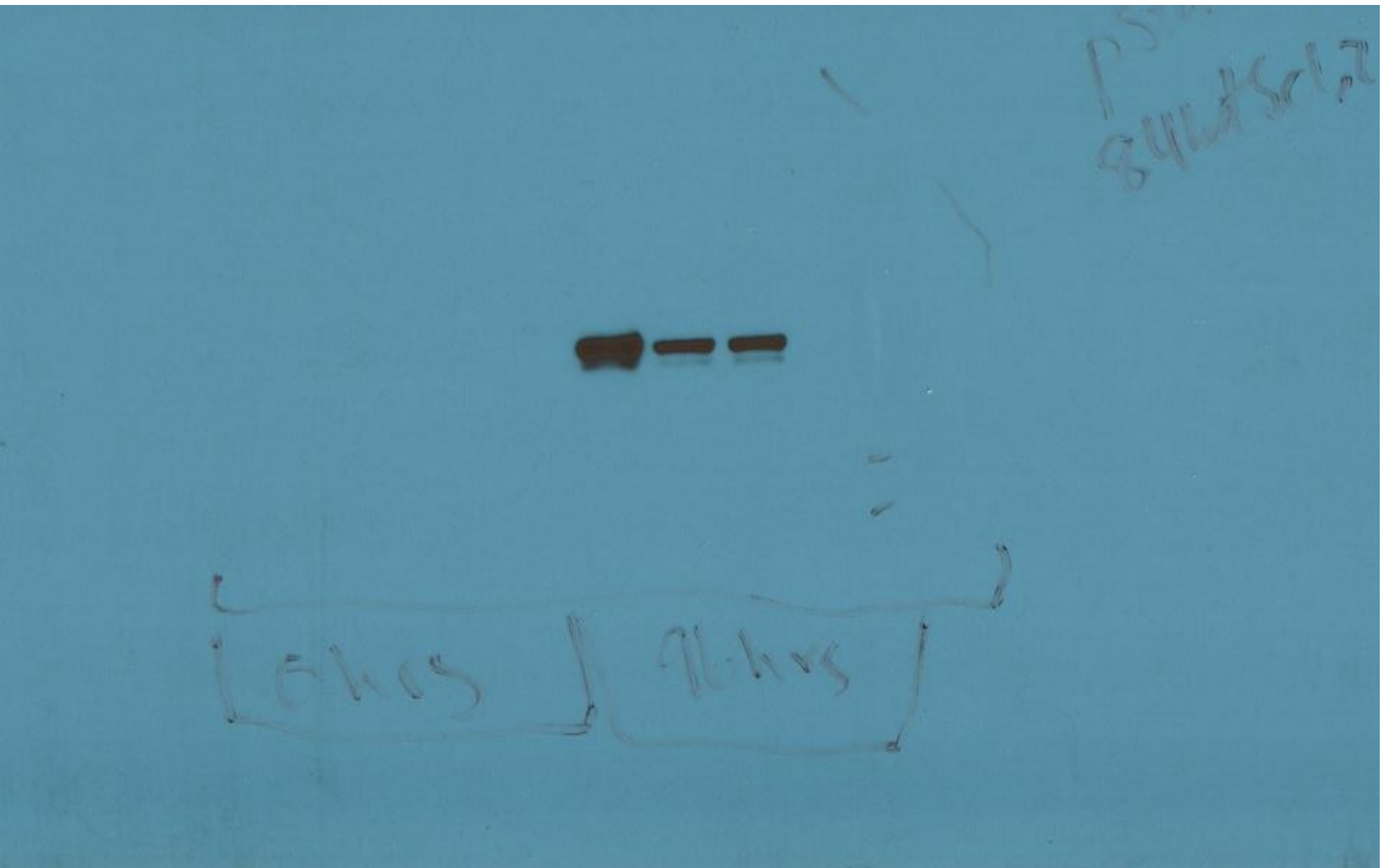

STAT1

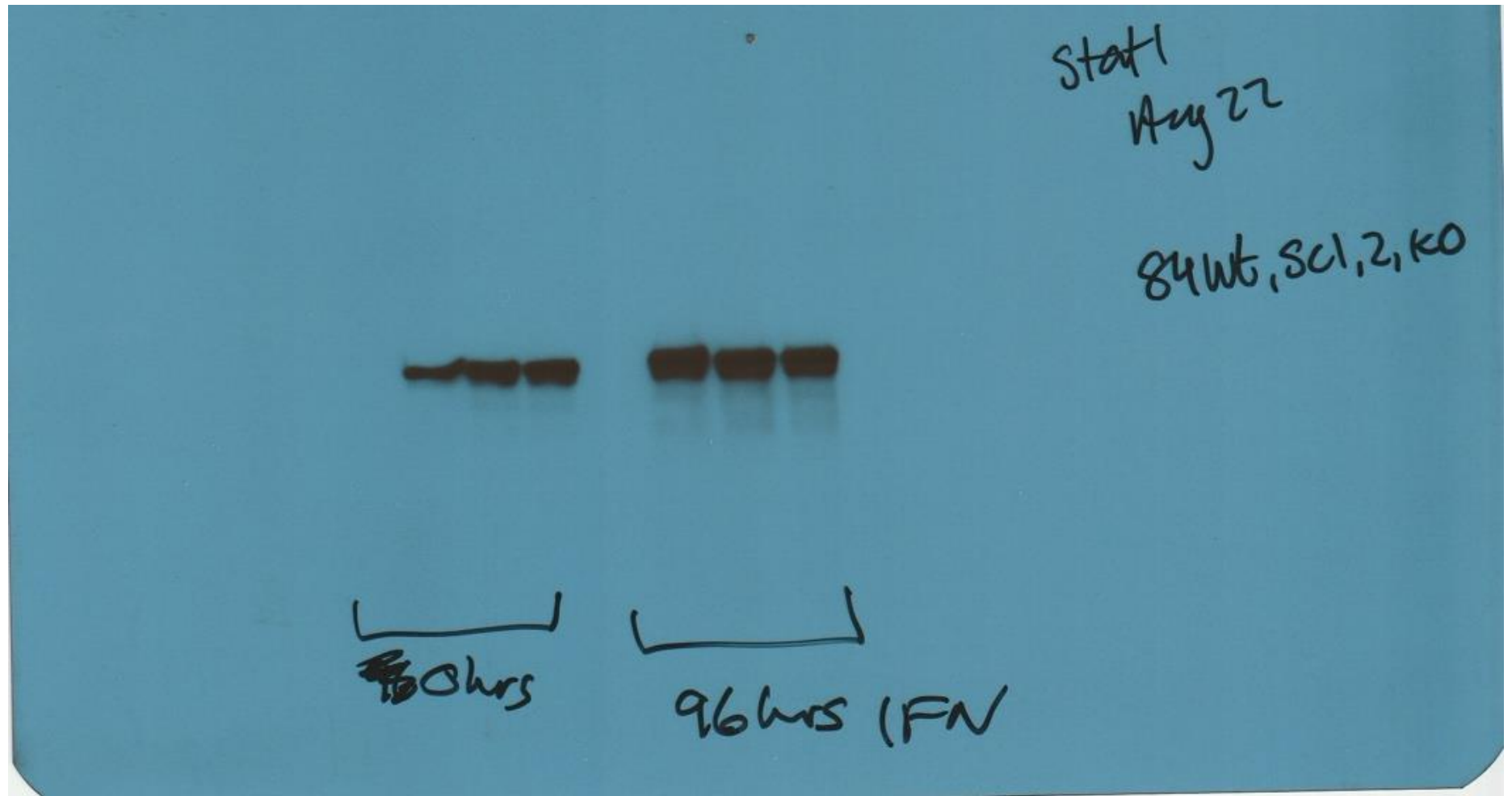

Beta actin

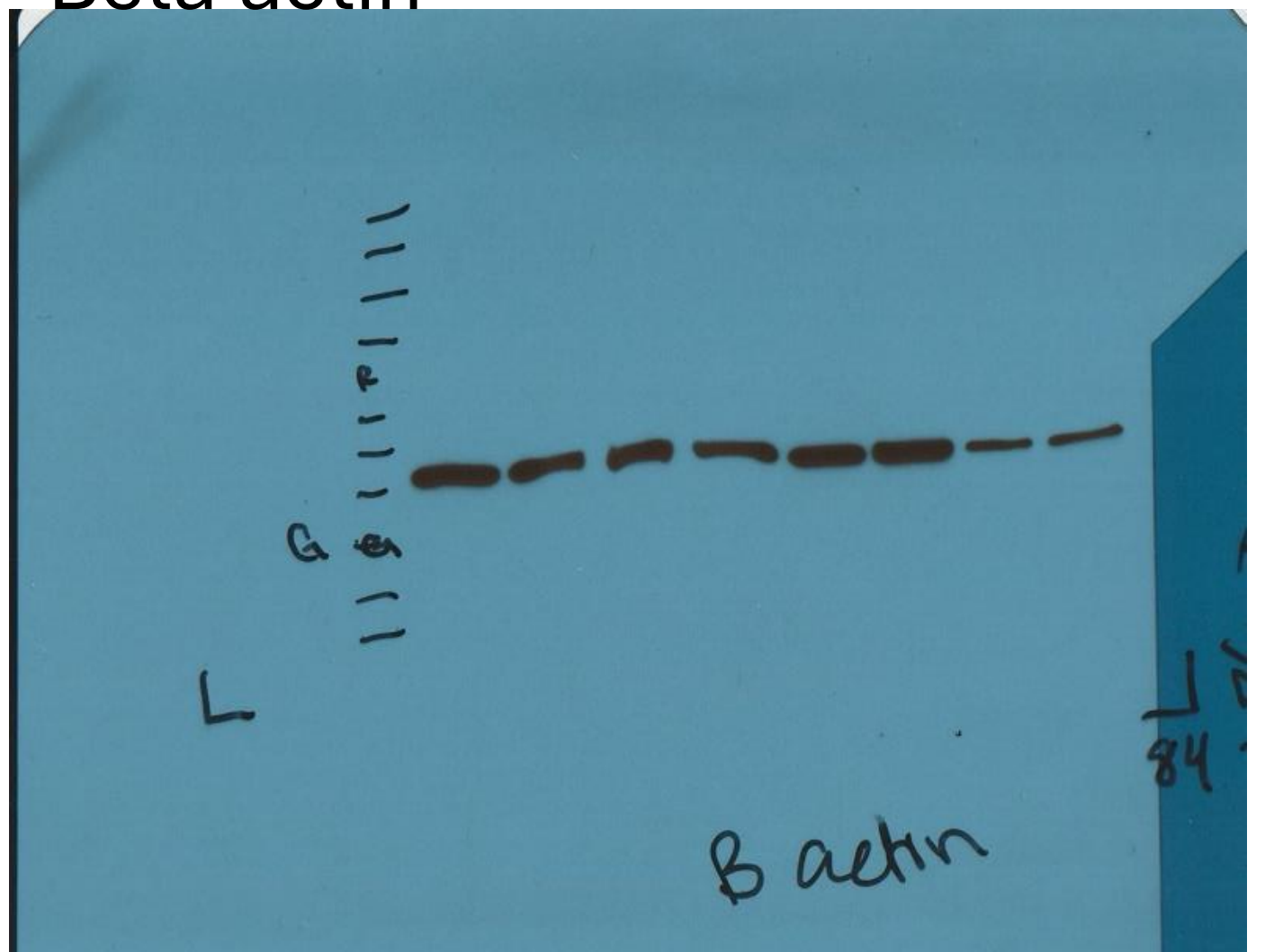

IHRT4606 supplementary figure 3A-1

Loading order (Left to right):

1. Ladder
2. WT 0ng/ml IFN-γ
3. WT 10ng/ml IFN-γ
4. STAT1 **knockout** 0ng/ml IFN-γ
5. STAT1 **knockout** 10ng/ml IFN-γ
6. STAT1 **KO+ empty vector** 10ng/ml IFN-γ
7. STAT1 **KO+ STAT1 Y701F**10ng/ml IFN-γ
8. STAT1 **KO+ STAT1 WT**10ng/ml IFN-γ
9. Ladder

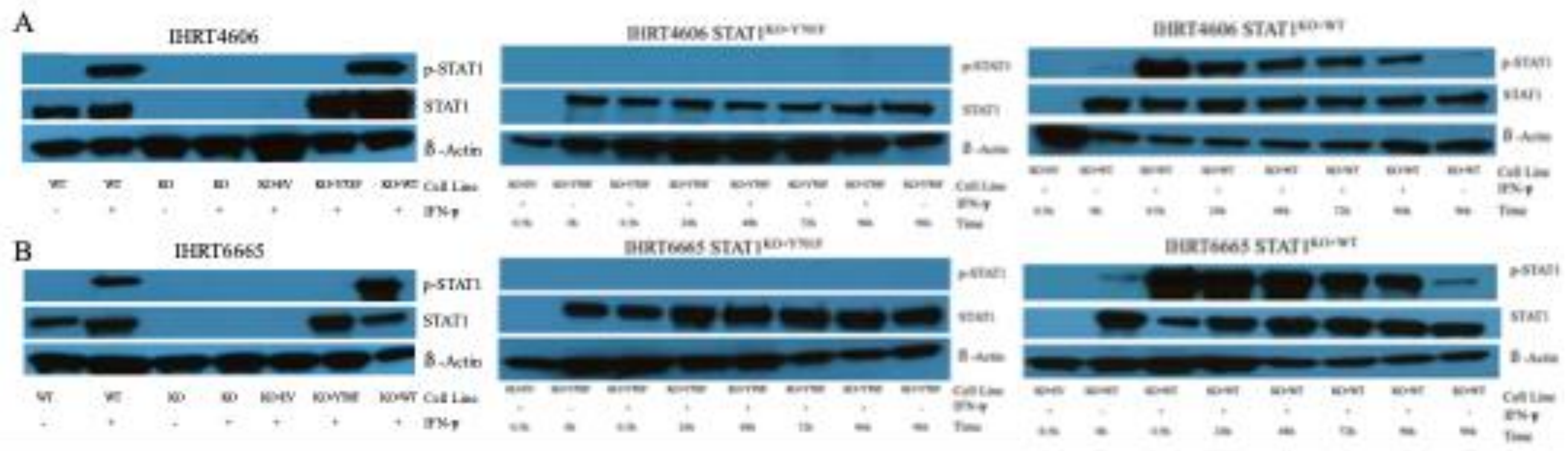

pSTAT1

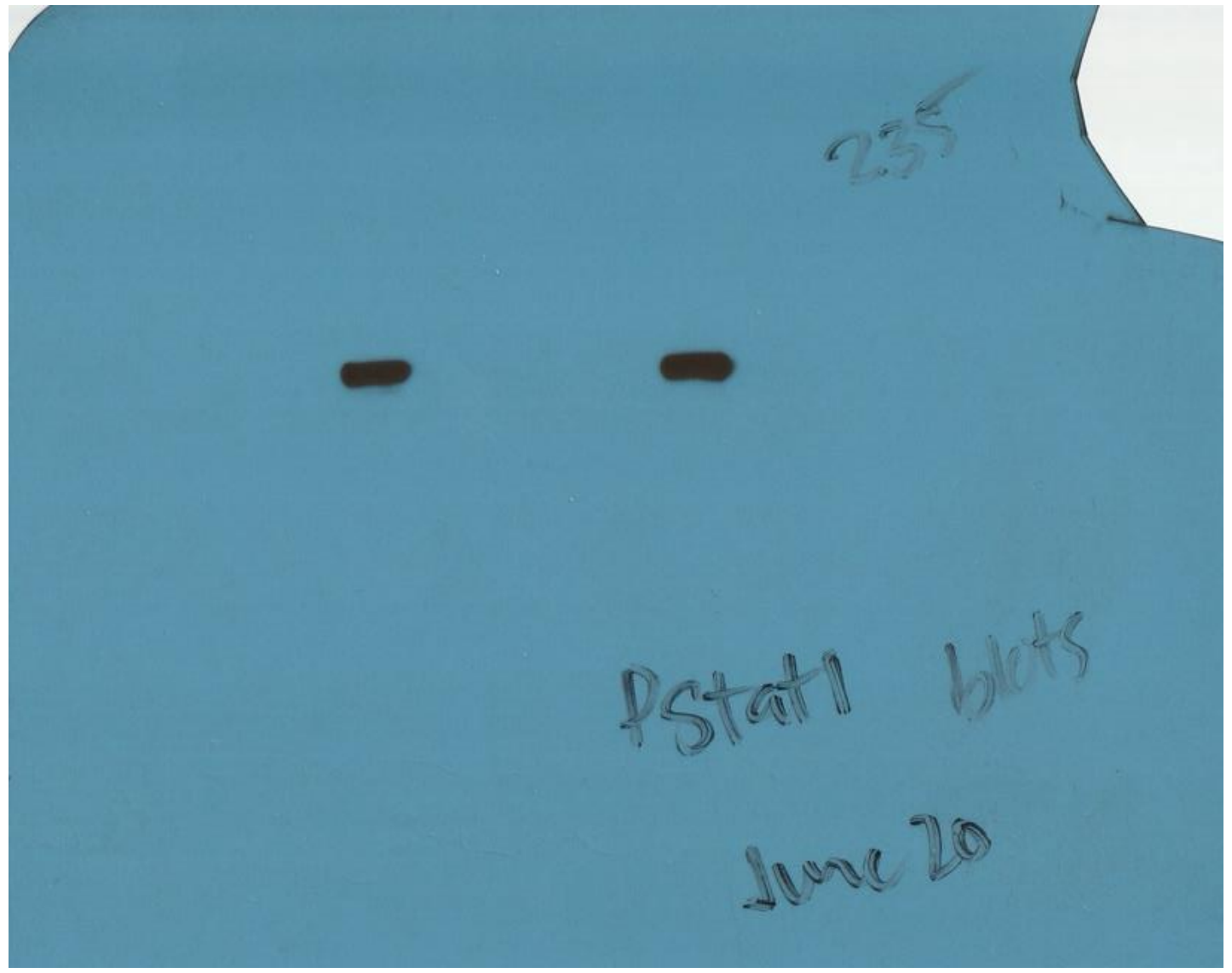

STAT1

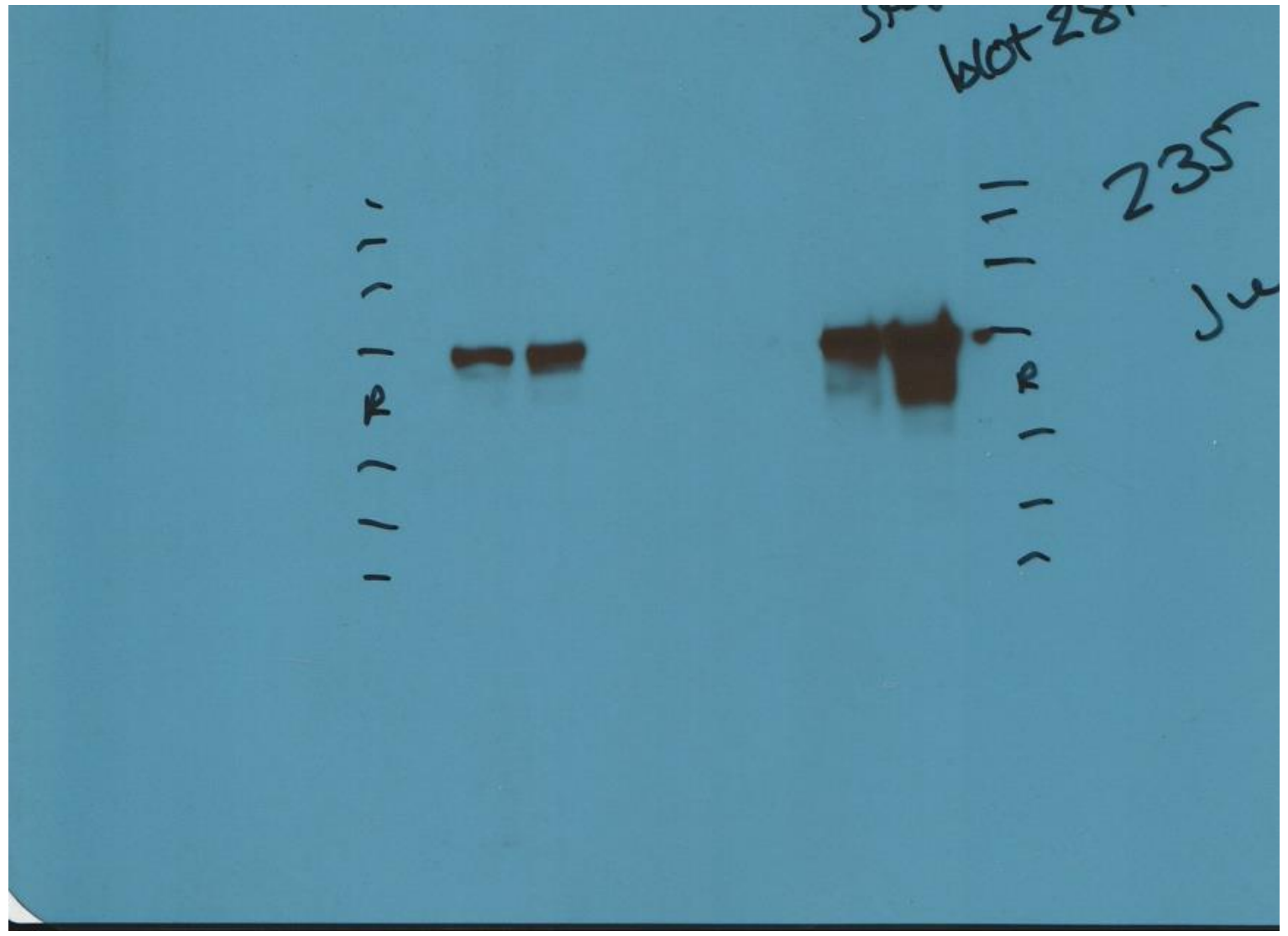

Beta actin

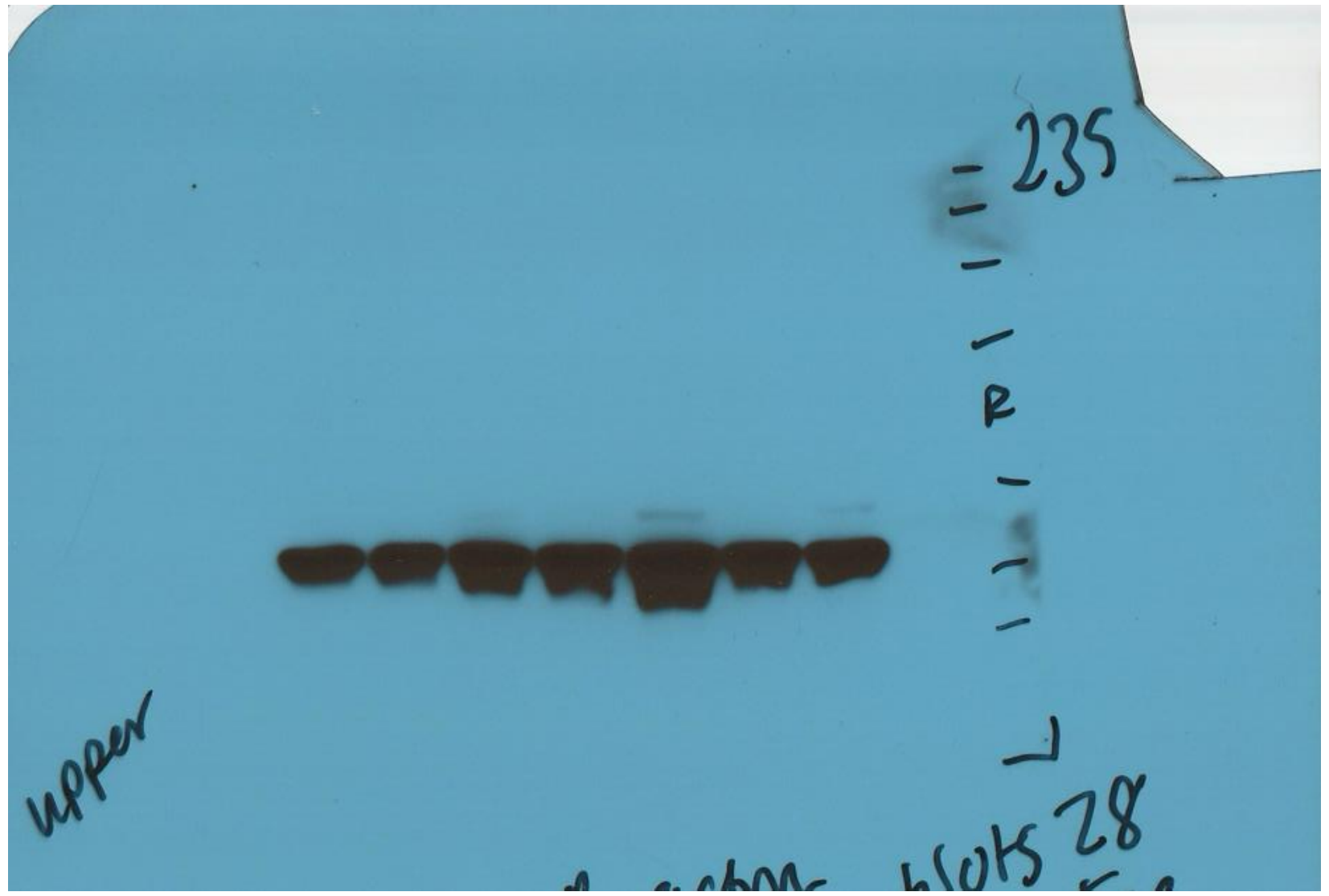

IHRT4606 supplementary figure 3A-2

Loading order (Left to right):

1. STAT1 **KO+ empty vector** 10ng/ml IFN-γ, 0.5h
2. STAT1 **KO+ STAT1 Y701F** 0ng/ml IFN-γ , 0h
3. STAT1 **KO+ STAT1 Y701F** 10ng/ml IFN-γ , 0.5h
4. STAT1 **KO+ STAT1 Y701F** 10ng/ml IFN-γ , 24h
5. STAT1 **KO+ STAT1 Y701F** 10ng/ml IFN-γ , 48h
6. STAT1 **KO+ STAT1 Y701F** 10ng/ml IFN-γ , 72h
7. STAT1 **KO+ STAT1 Y701F** 10ng/ml IFN-γ , 96h
8. STAT1 **KO+ STAT1 Y701F** 0ng/ml IFN-γ , 96h
9. ladder
10. empty

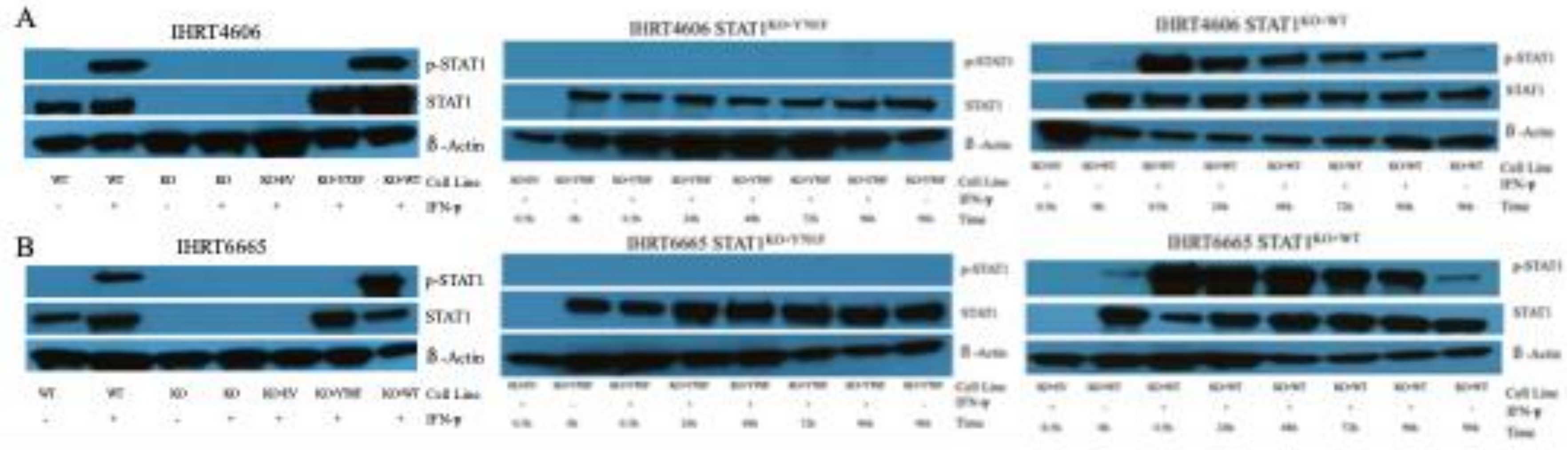

pSTAT1

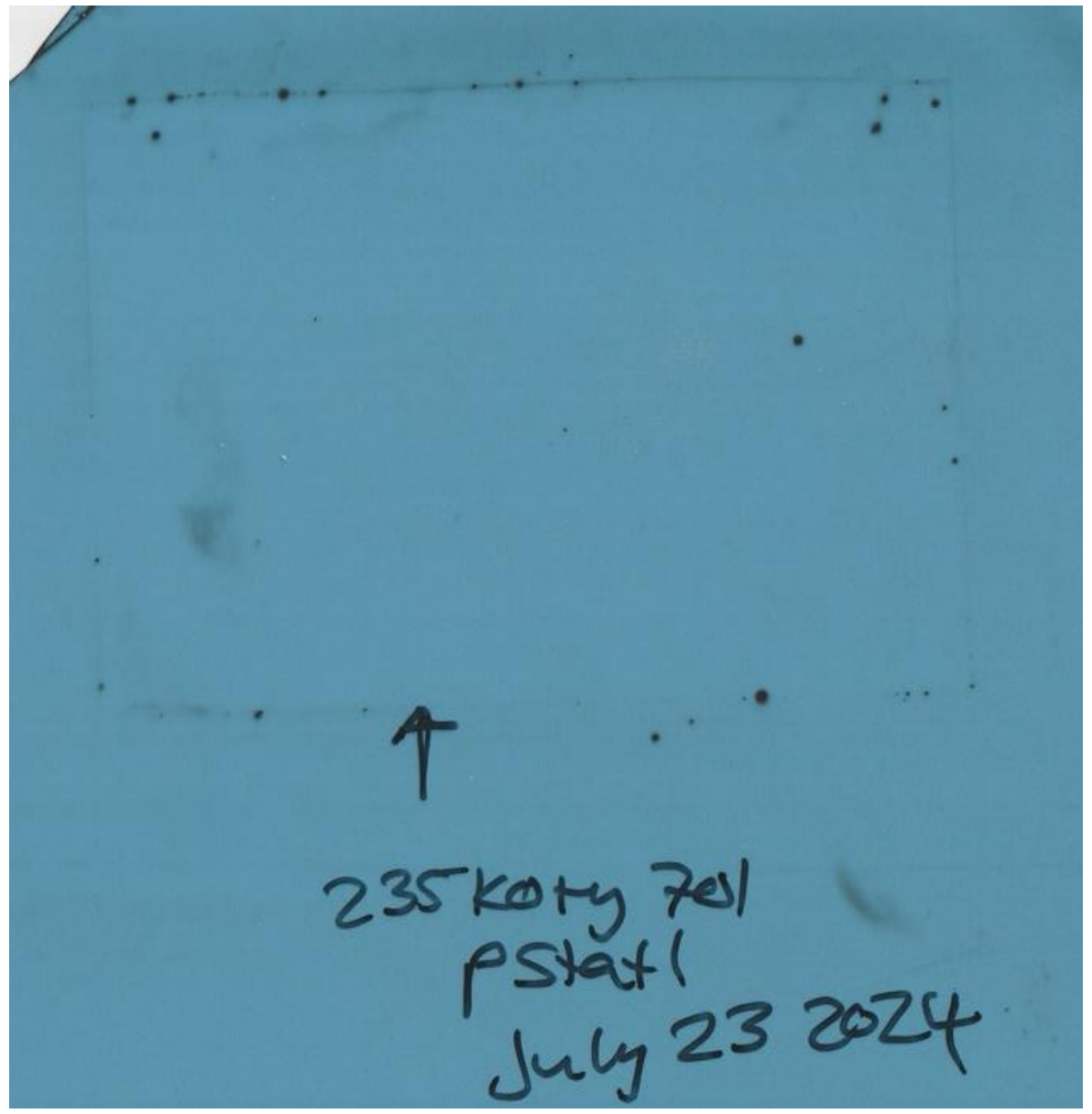

STAT1

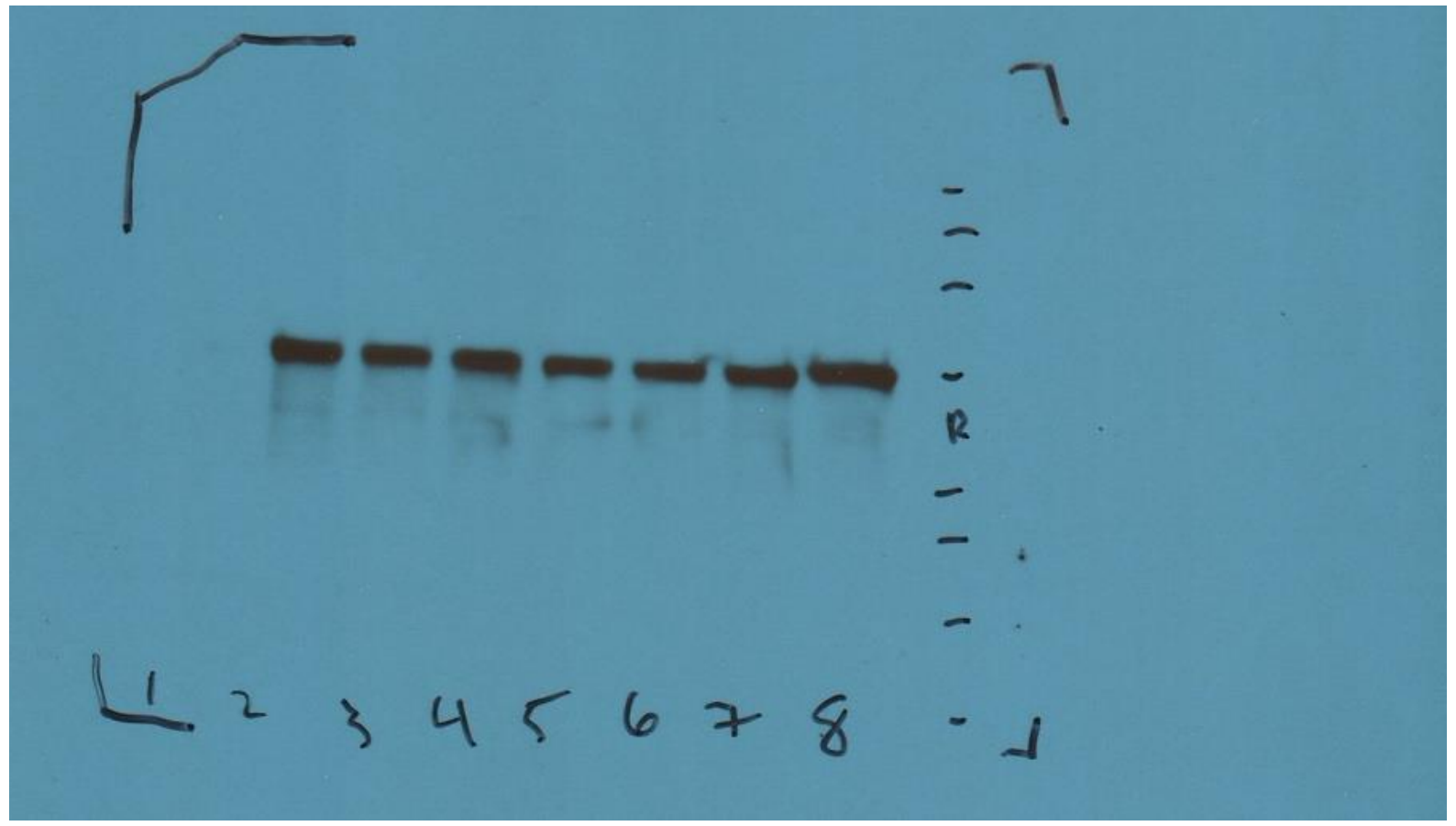

Beta actin

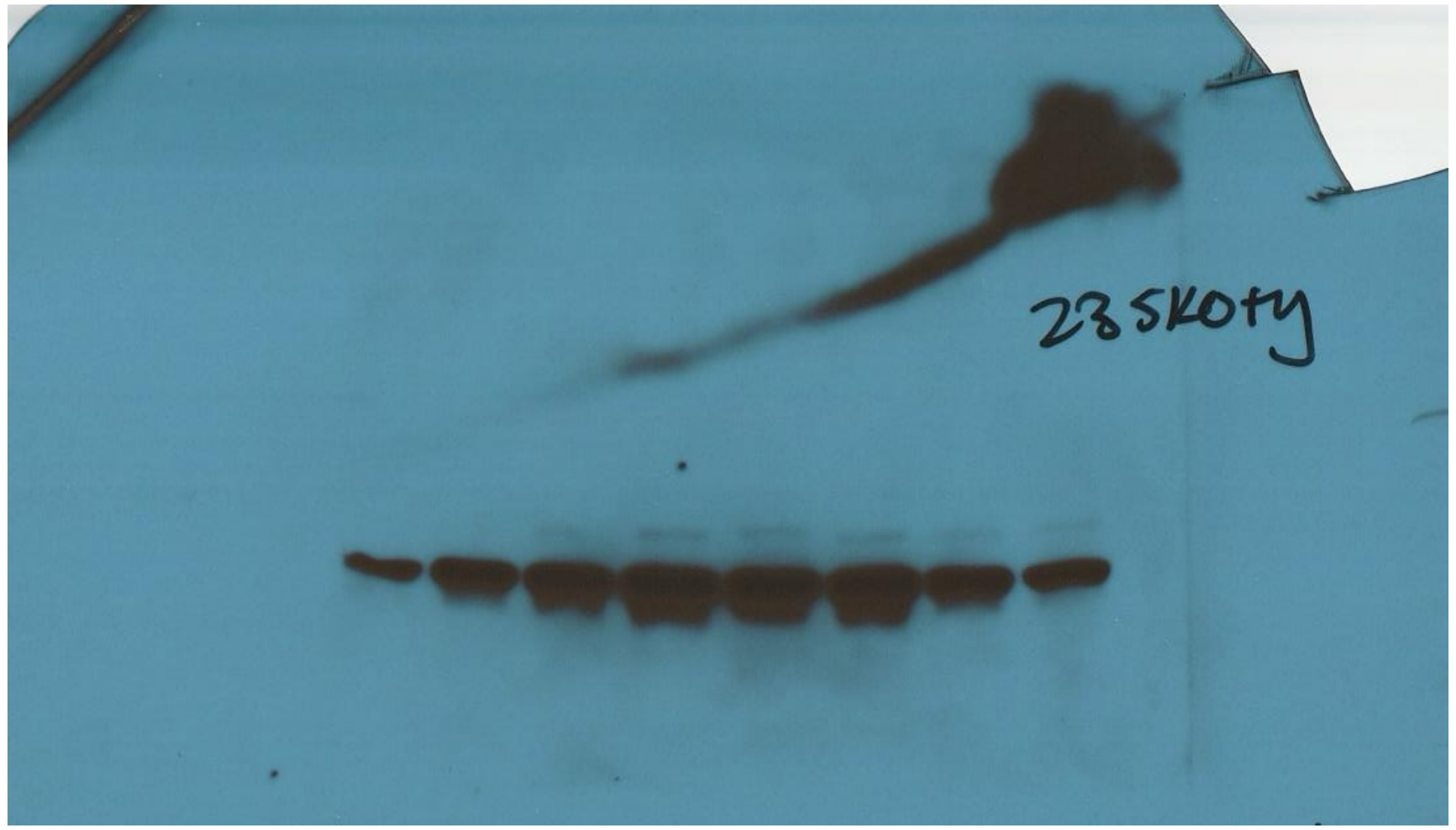

IHRT4606 supplementary figure 3A-3

Loading order (Left to right):

1. STAT1 **KO+ empty vector** 10ng/ml IFN-γ, 0.5h
2. STAT1 **KO+ STAT1 WT** 0ng/ml IFN-γ , 0h
3. STAT1 **KO+ STAT1 WT** 10ng/ml IFN-γ , 0.5h
4. STAT1 **KO+ STAT1 WT** 10ng/ml IFN-γ , 24h
5. STAT1 **KO+ STAT1 WT** 10ng/ml IFN-γ , 48h
6. STAT1 **KO+ STAT1 WT** 10ng/ml IFN-γ , 72h
7. STAT1 **KO+ STAT1 WT** 10ng/ml IFN-γ , 96h
8. STAT1 **KO+ STAT1 WT 0**ng/ml IFN-γ , 96h
9. ladder
10. empty

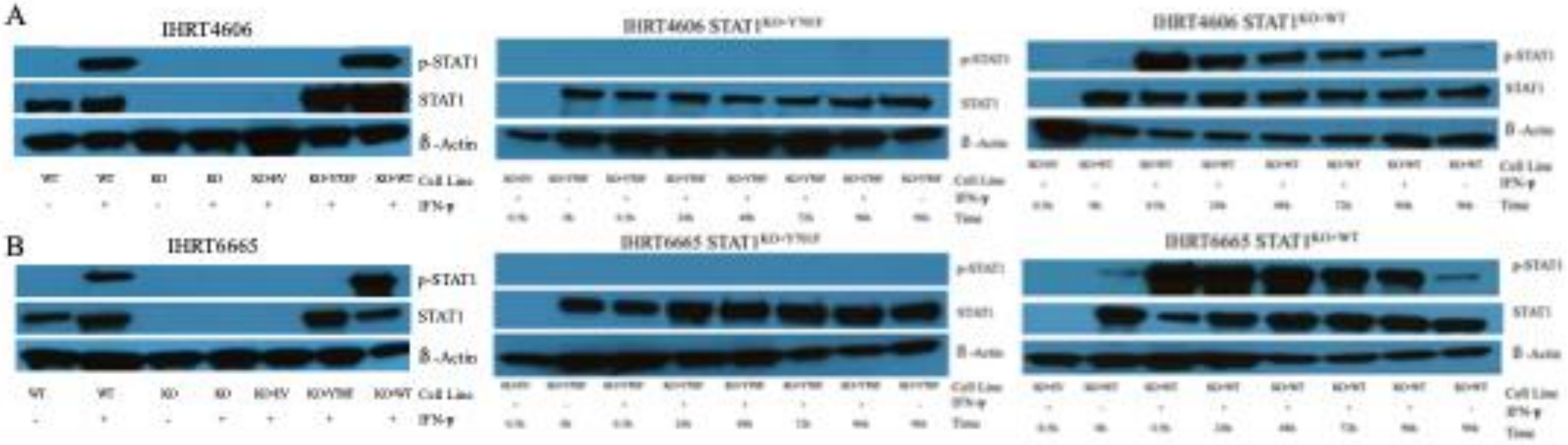

pSTAT1

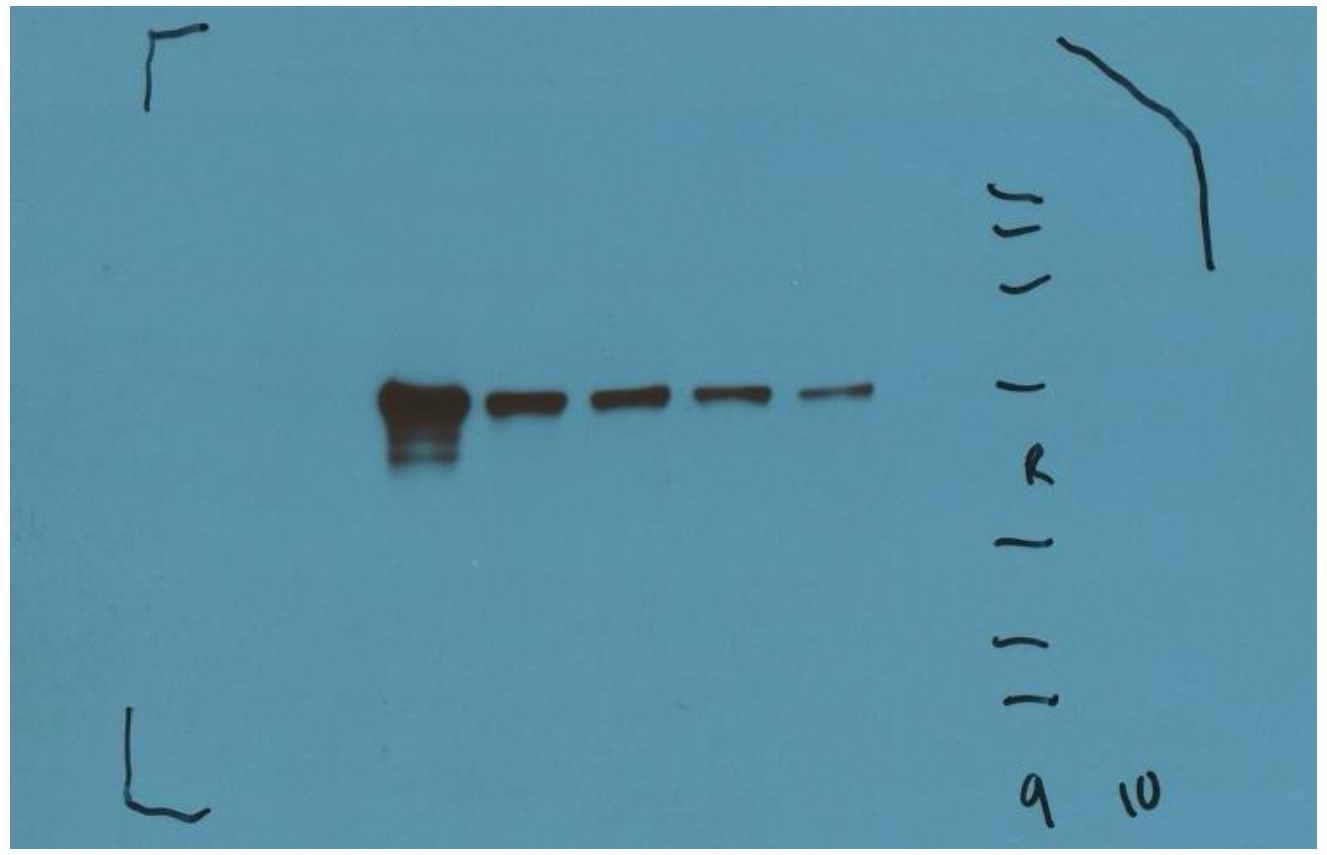

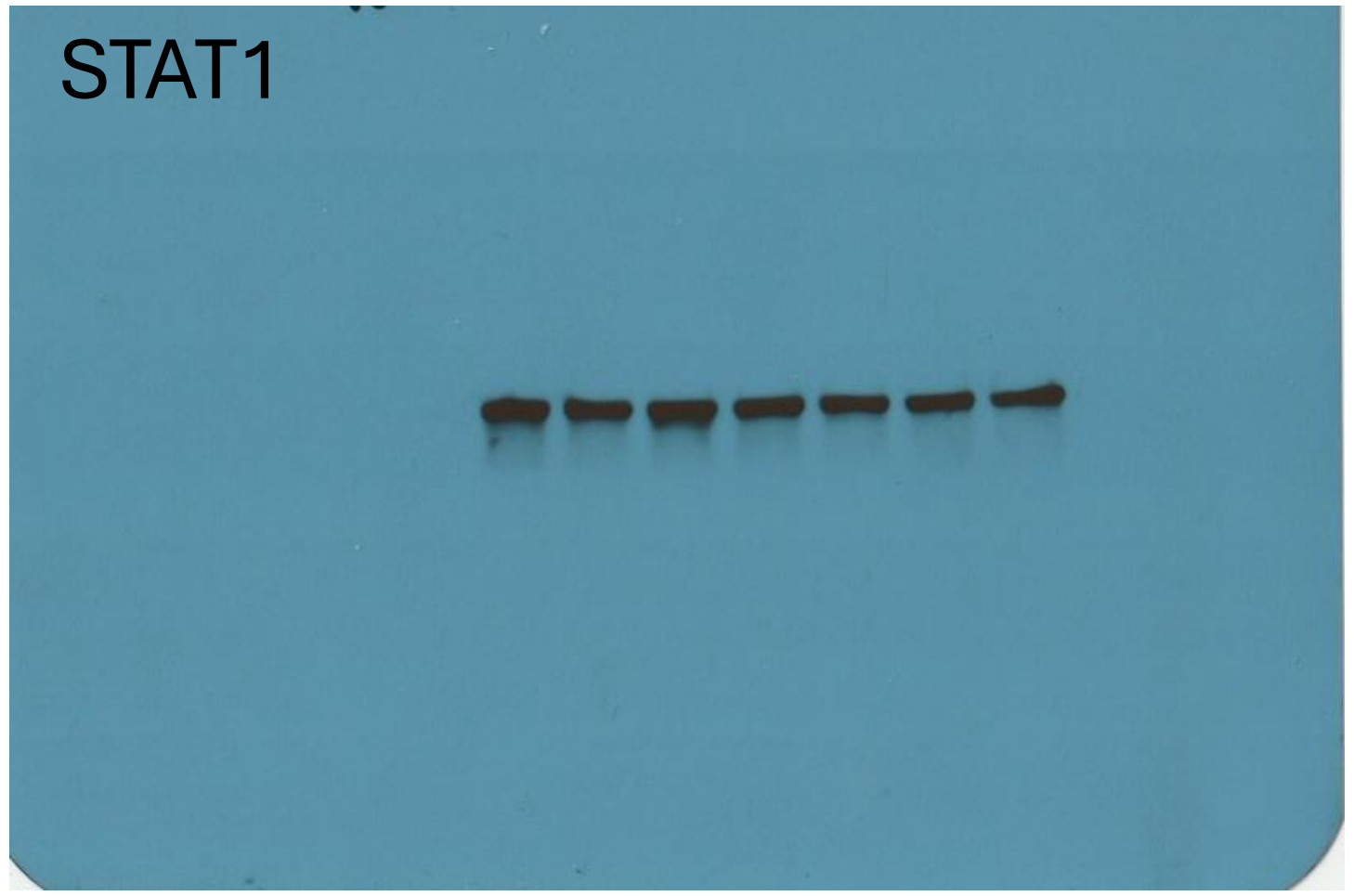

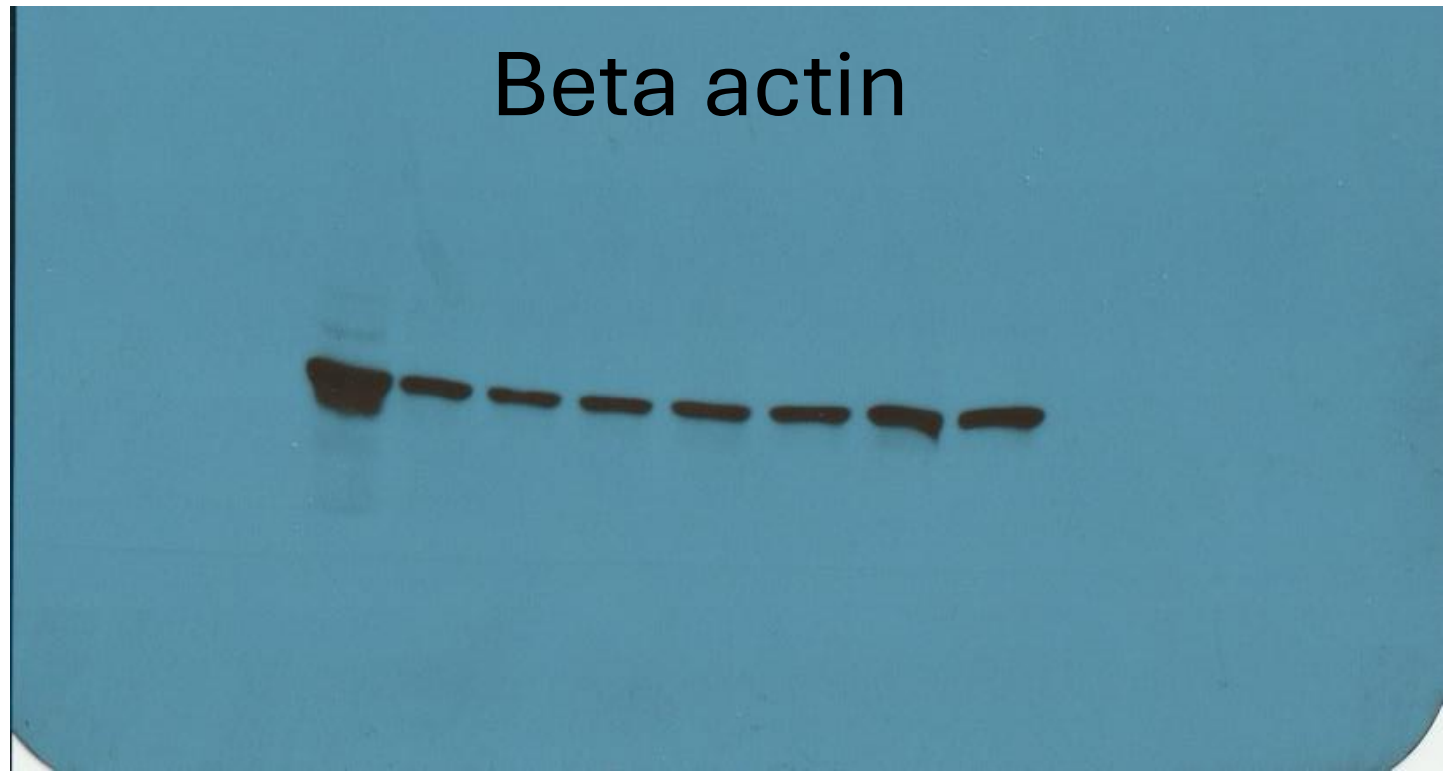

IHRT6665 supplementary figure 3B-1

Loading order (Left to right):

1. Ladder
2. WT 0ng/ml IFN-γ
3. WT 10ng/ml IFN-γ
4. STAT1 **knockout** 0ng/ml IFN-γ
5. STAT1 **knockout** 10ng/ml IFN-γ
6. STAT1 **KO+ empty vector** 10ng/ml IFN-γ
7. STAT1 **KO+ STAT1 Y701F**10ng/ml IFN-γ
8. STAT1 **KO+ STAT1 WT**10ng/ml IFN-γ
9. empty
10. empty

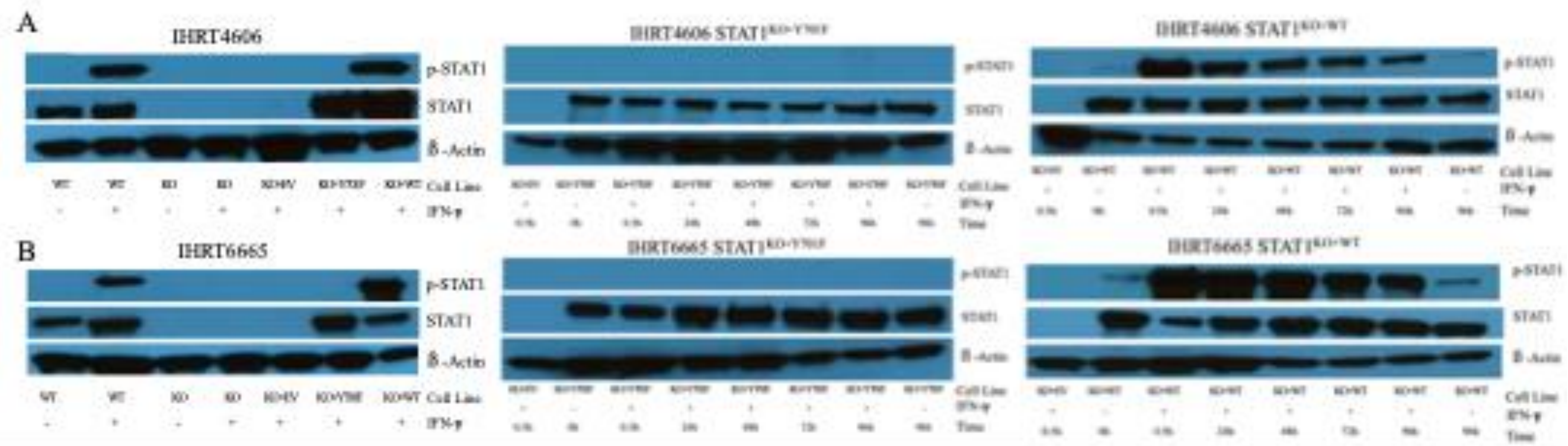

pSTAT1

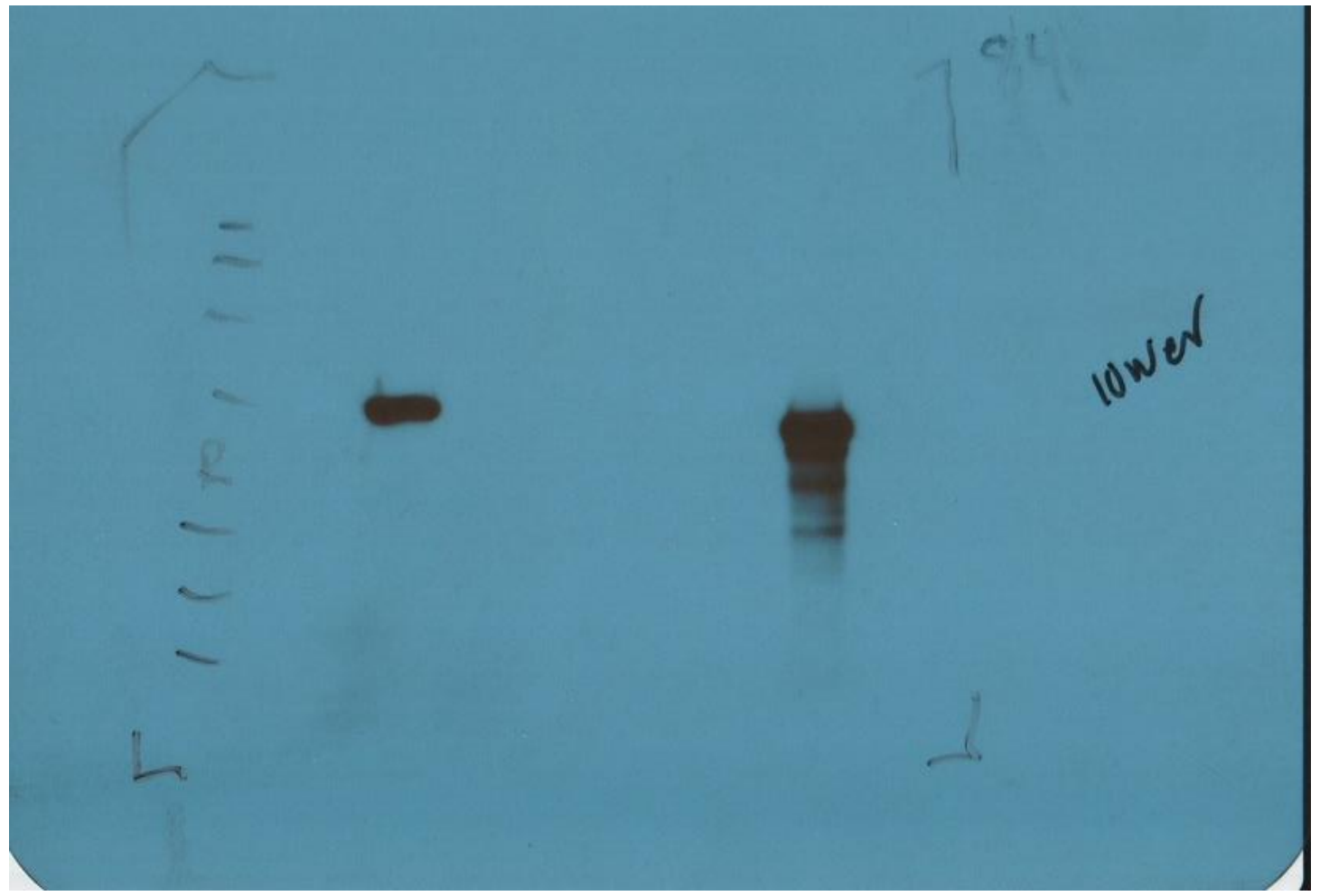

STAT1

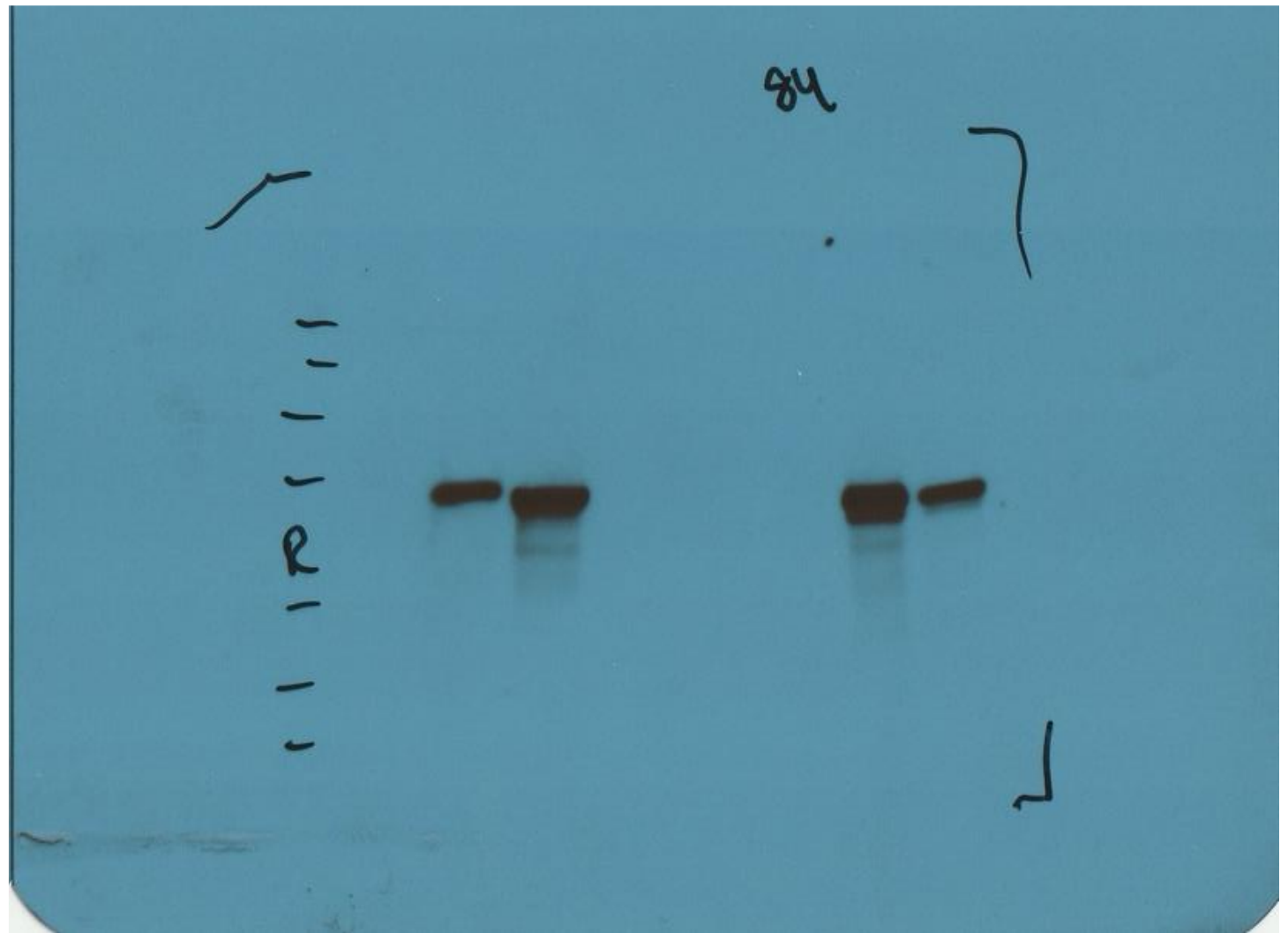

Beta actin

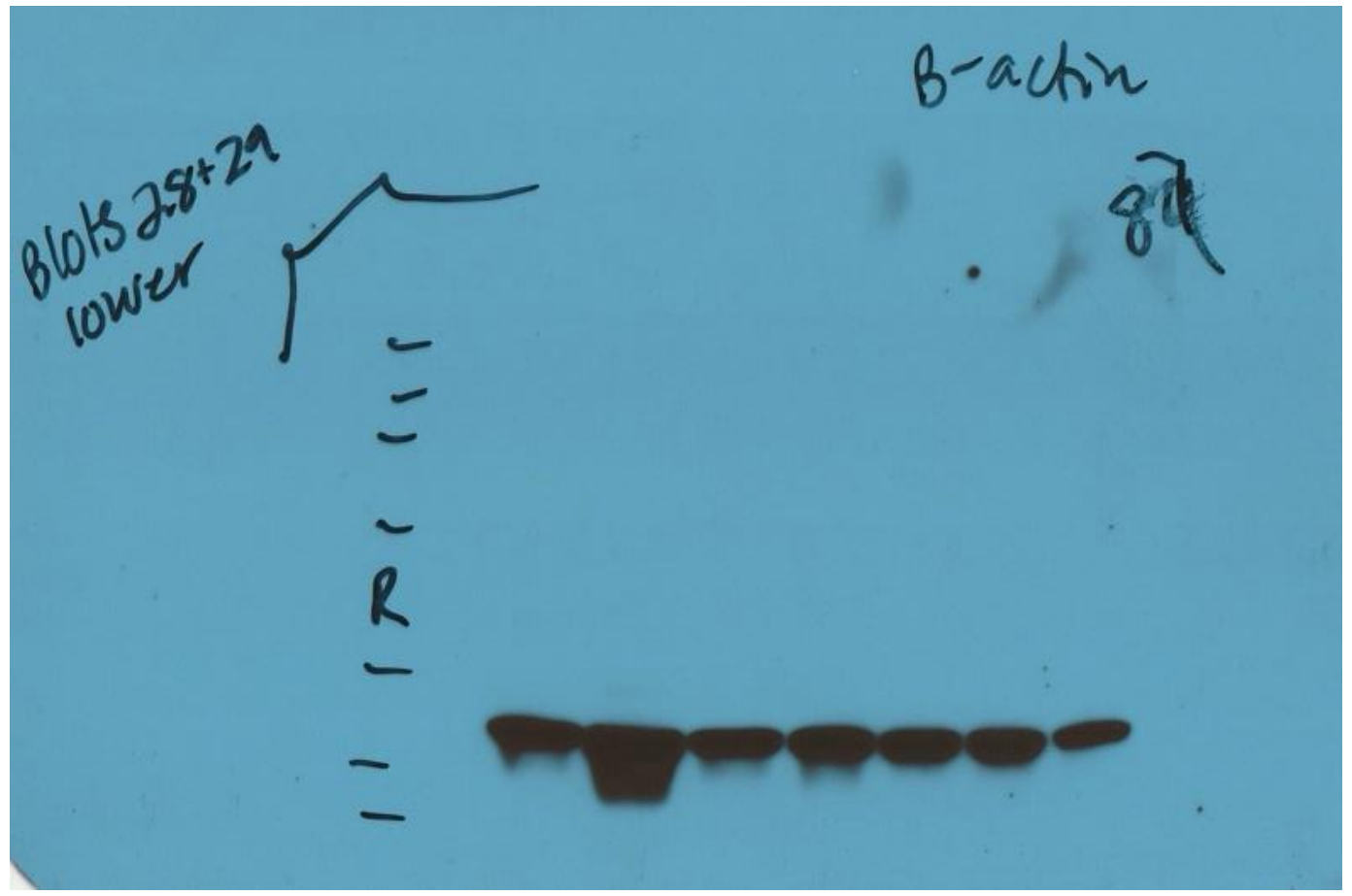

IHRT6665 supplementary figure 3B-2

Loading order:

1. STAT1 **KO+ empty vector** 10ng/ml IFN-γ, 0.5
2. STAT1 **KO+ STAT1 Y701F** 0ng/ml IFN-γ , 0h
3. STAT1 **KO+ STAT1 Y701F** 10ng/ml IFN-γ , 0.5h
4. STAT1 **KO+ STAT1 Y701F** 10ng/ml IFN-γ , 24h
5. STAT1 **KO+ STAT1 Y701F** 10ng/ml IFN-γ , 48h
6. STAT1 **KO+ STAT1 Y701F** 10ng/ml IFN-γ , 72h
7. STAT1 **KO+ STAT1 Y701F** 10ng/ml IFN-γ , 96h
8. STAT1 **KO+ STAT1 Y701F** 0ng/ml IFN-γ , 96h
9. ladder
10. empty

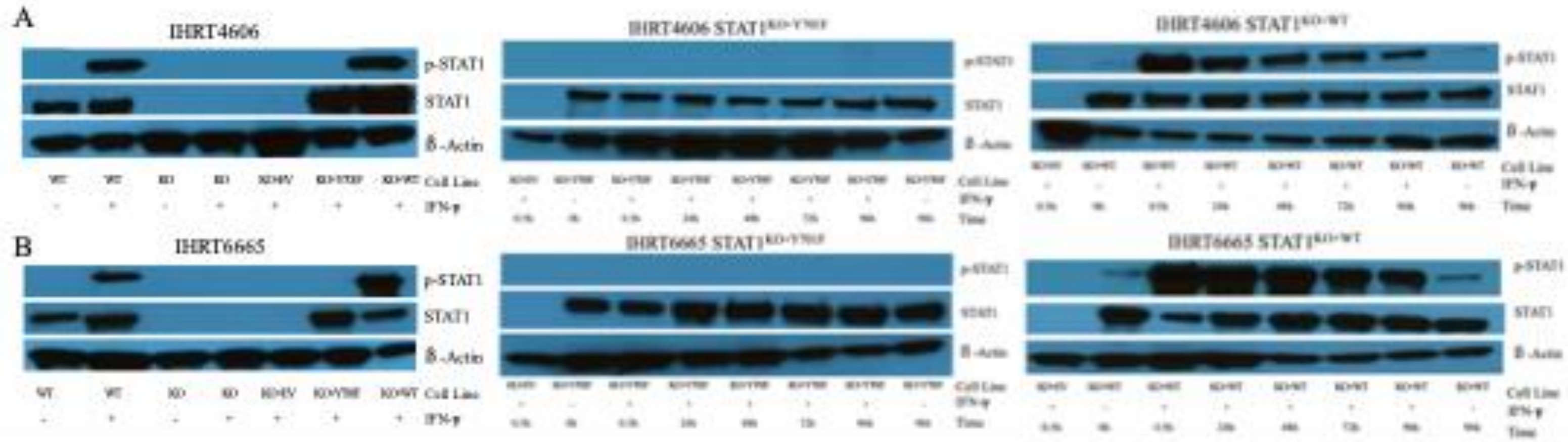

pSTAT1

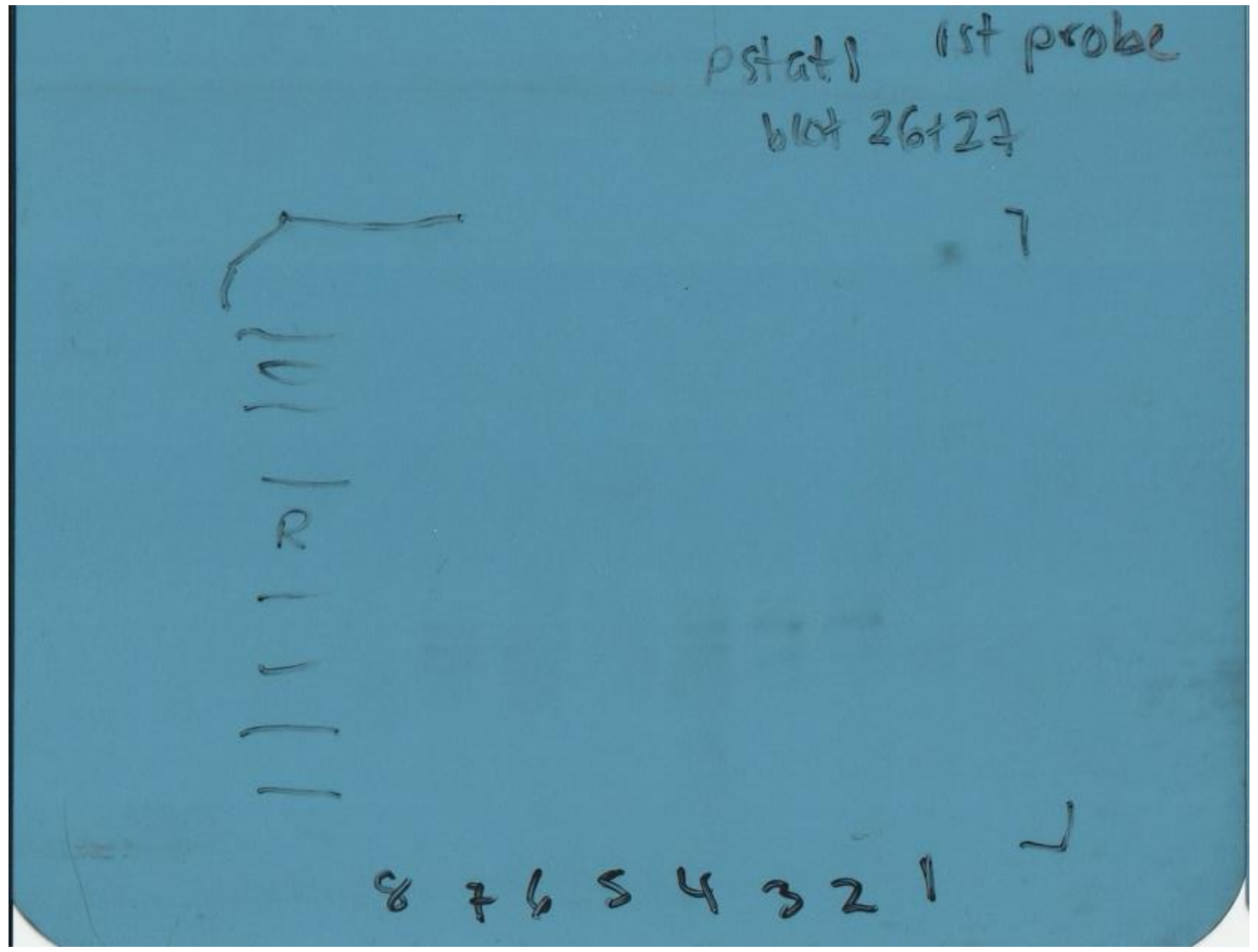

STAT1

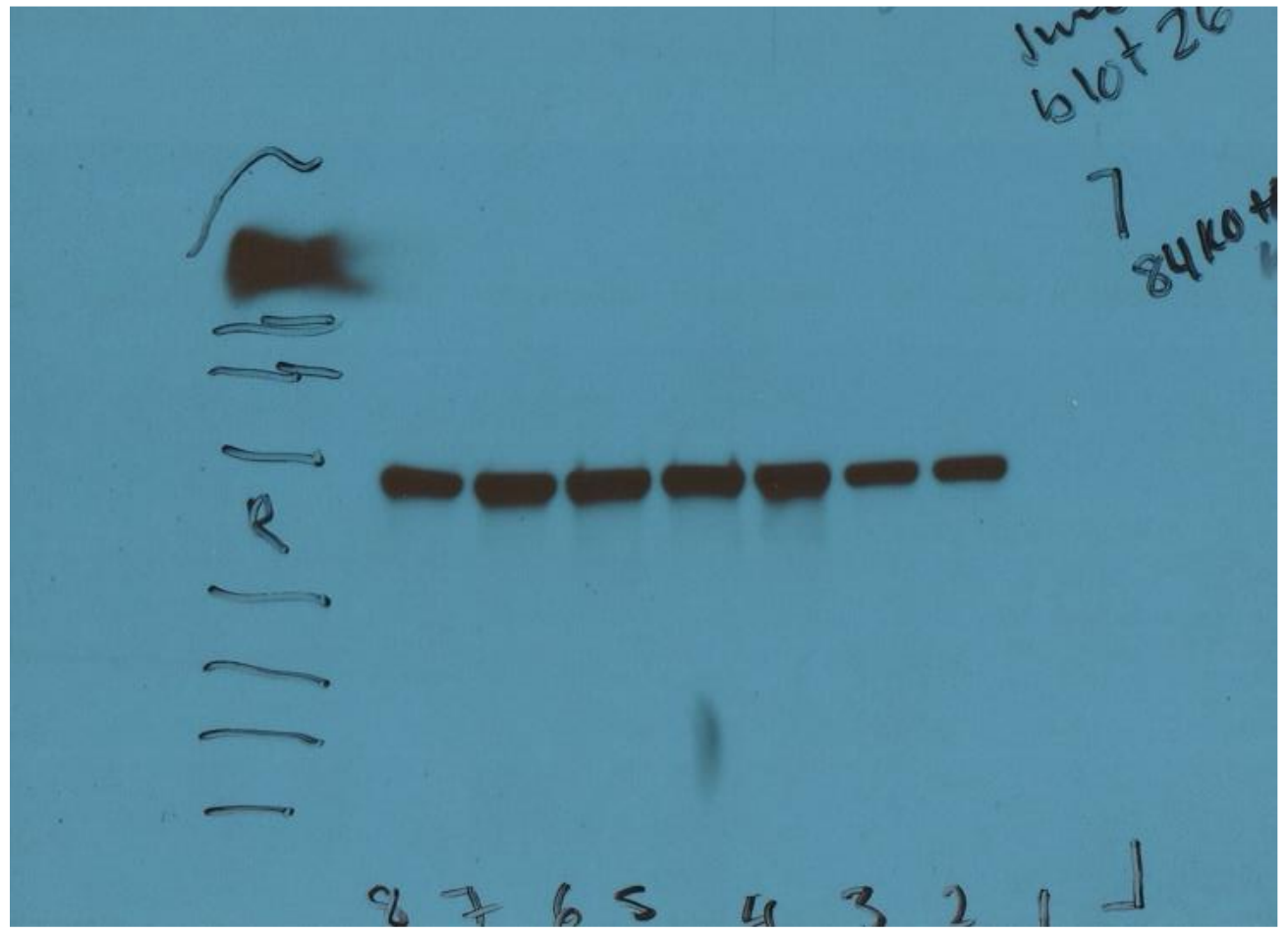

Beta actin

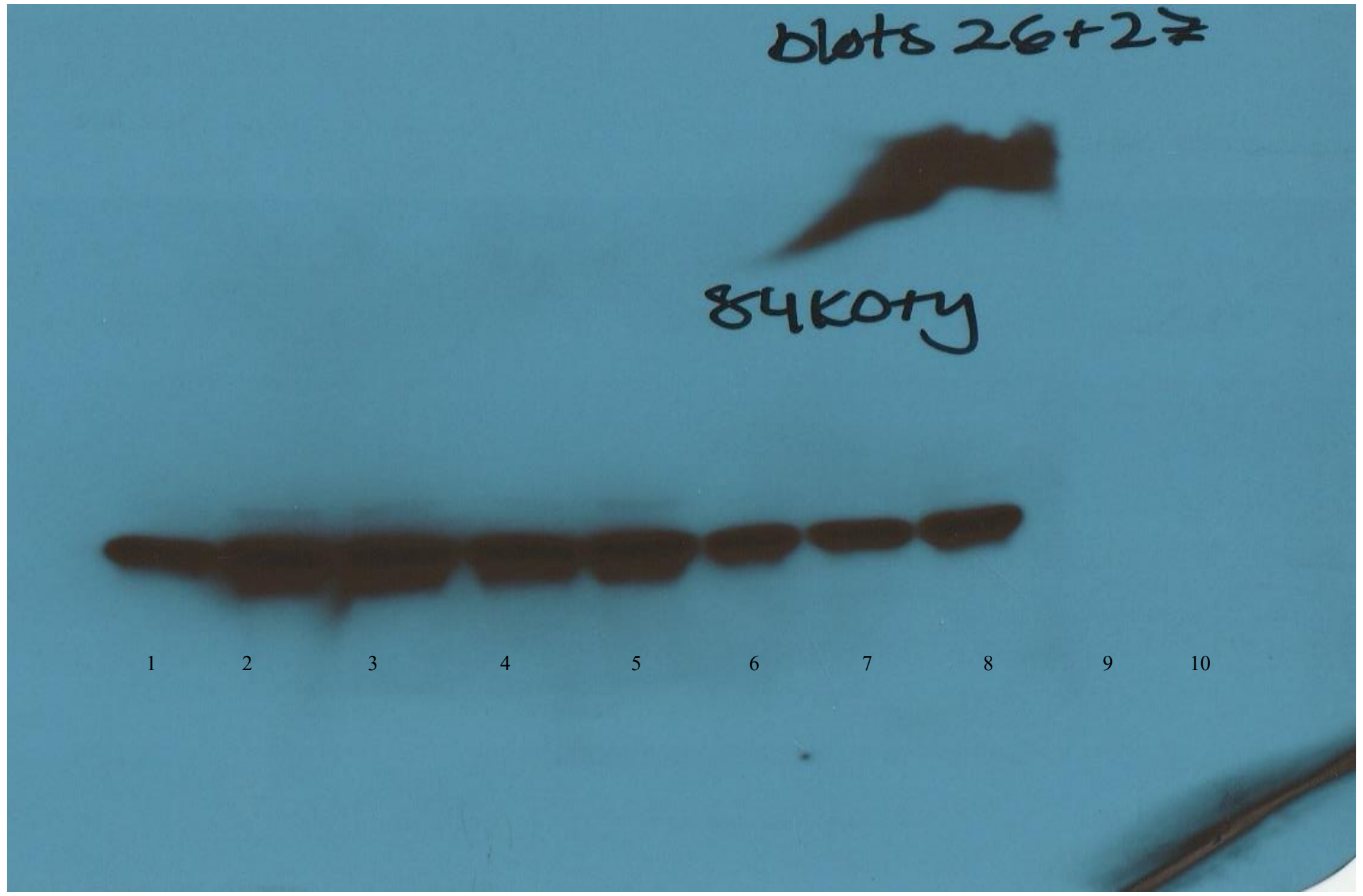

IHRT6665 supplementary figure 3B-3

Loading order (Left to right):

1. STAT1 **KO+ empty vector** 10ng/ml IFN-γ, 0.5h
2. STAT1 **KO+ STAT1 WT** 0ng/ml IFN-γ , 0h
3. STAT1 **KO+ STAT1 WT** 10ng/ml IFN-γ , 0.5h
4. STAT1 **KO+ STAT1 WT** 10ng/ml IFN-γ , 24h
5. STAT1 **KO+ STAT1 WT** 10ng/ml IFN-γ , 48h
6. STAT1 **KO+ STAT1 WT** 10ng/ml IFN-γ , 72h
7. STAT1 **KO+ STAT1 WT** 10ng/ml IFN-γ , 96h
8. STAT1 **KO+ STAT1 WT** 0ng/ml IFN-γ , 96h
9. empty
10. ladder

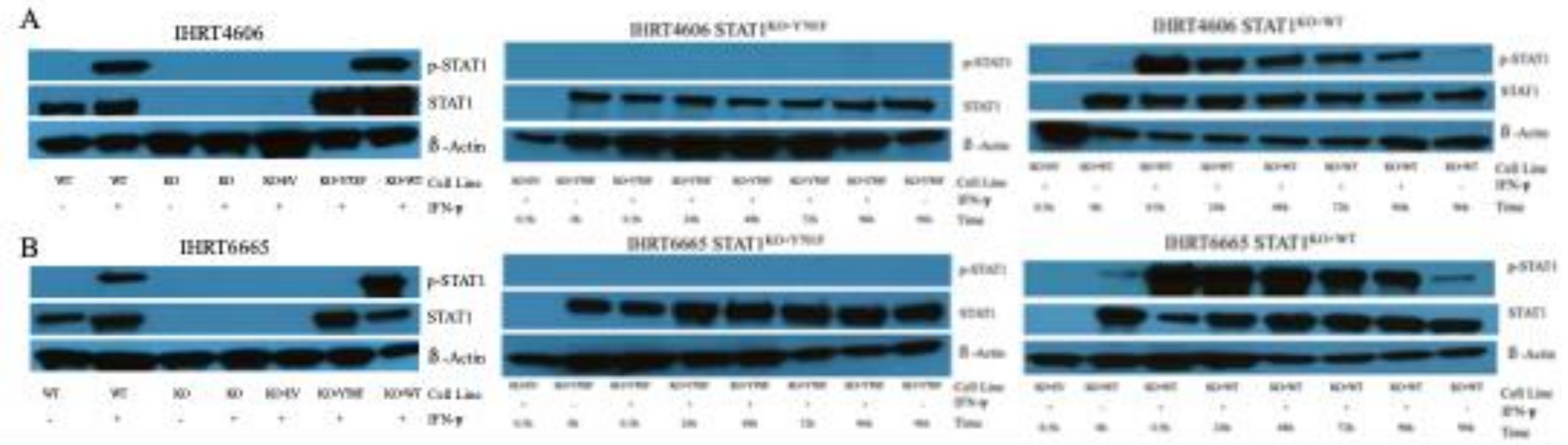

pSTAT1

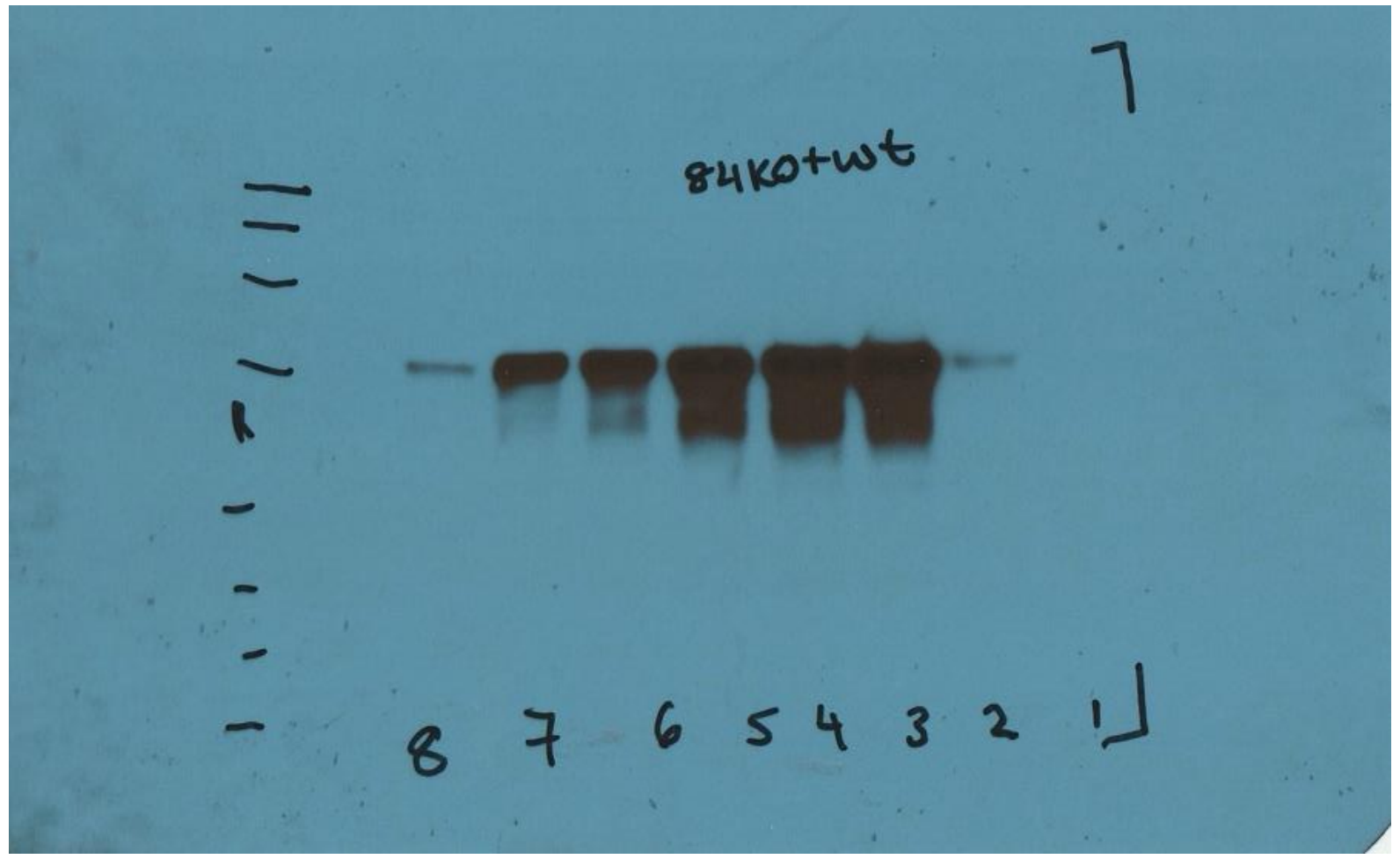

STAT1

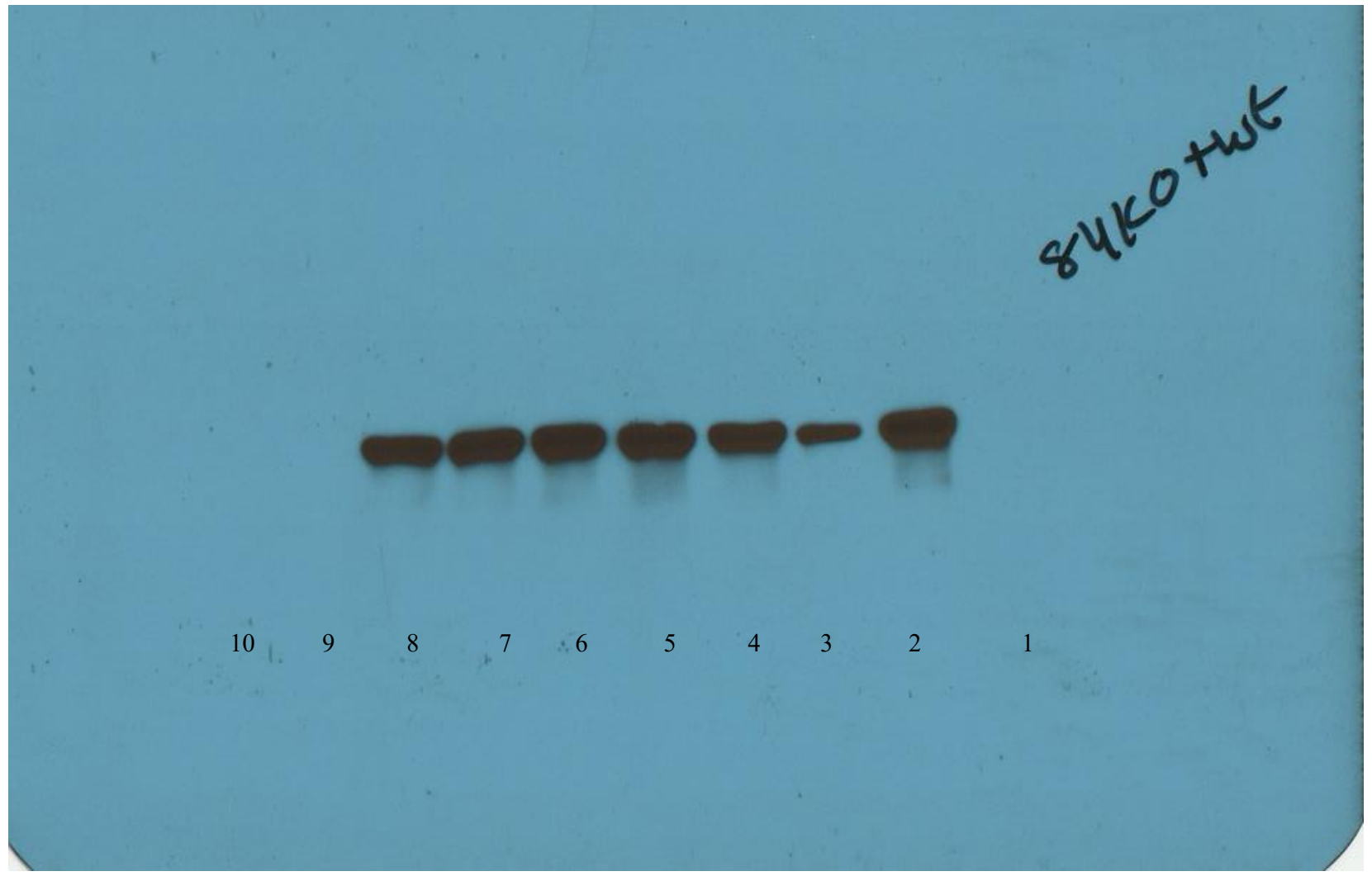

Beta actin

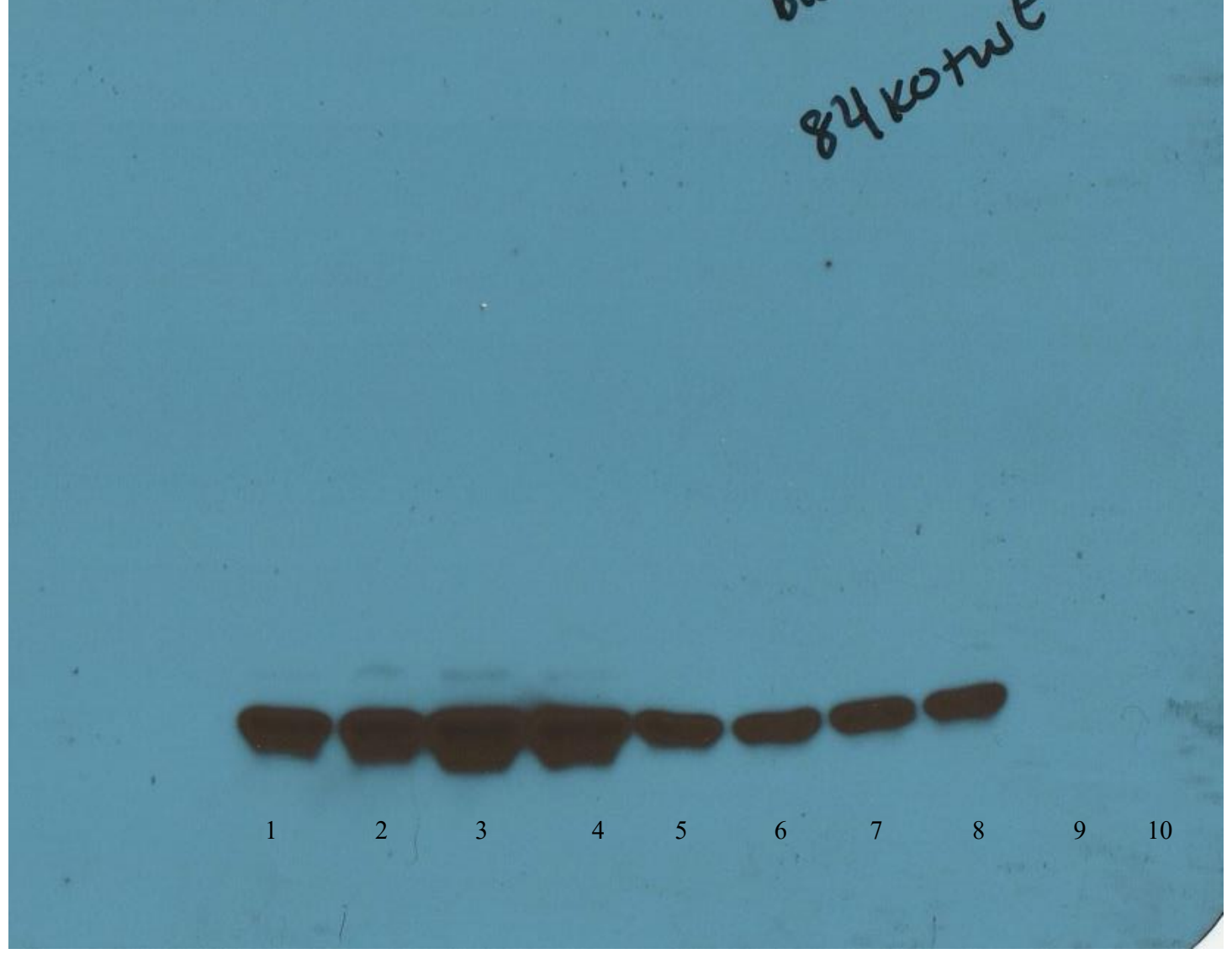

Supplement: Supplementary file 6 [file pone.0361295.s011.pdf]
